# Novel Perspective on Alzheimer’s Disease Treatment: Rosmarinic Acid Molecular Interplay with Copper(II) and Amyloid β

**DOI:** 10.3390/life10070118

**Published:** 2020-07-20

**Authors:** Arian Kola, Aleksandra Hecel, Stefania Lamponi, Daniela Valensin

**Affiliations:** 1 Department of Biotechnology, Chemistry and Pharmacy, University of Siena, Via Aldo Moro 2, 53100 Siena, Italy; kola2@student.unisi.it (A.K.); stefania.lamponi@unisi.it (S.L.); 2 Faculty of Chemistry, University of Wroclaw, F. Joliot Curie 14, 50383 Wroclaw, Poland; aleksandra.hecel@chem.uni.wroc.pl

**Keywords:** amyloid β, copper, rosmarinic acid, antioxidant, polyphenols

## Abstract

Alzheimer’s disease is a severe disorder that affects millions of people worldwide. It is a very debilitating disease with no cure at the moment. The necessity of finding an effective treatment is very demanding, and the entire scientific community is putting in a lot of effort to address this issue. The major hallmark of Alzheimer’s disease is the presence of toxic aggregated species in the brain, impaired metal homeostasis, and high levels of oxidative stress. Rosmarinic acid is a well-known potent antioxidant molecule, the efficacy of which has been proved both in vitro and in vivo. In this study, we investigated the possible role played by rosmarinic acid as a mediator of the copper(II)-induced neurotoxicity. Several spectroscopic techniques and biological assays were applied to characterize the metal complexes and to evaluate the cytotoxicity and the mutagenicity of rosmarinic acid and its Cu(II) complex. Our data indicate that rosmarinic acid is able to interfere with the interaction between amyloid β and Cu(II) by forming an original ternary association.

## 1. Introduction

The therapeutic properties of plants are well known starting with the ancient civilizations in Mesopotamia, Egypt, India, China, South America, and in the Mediterranean area. Herbal medicine is a traditional science based on the knowledge of plant composition and of the preparation methods specific for any herbal formula [1].

**Scheme 1:**
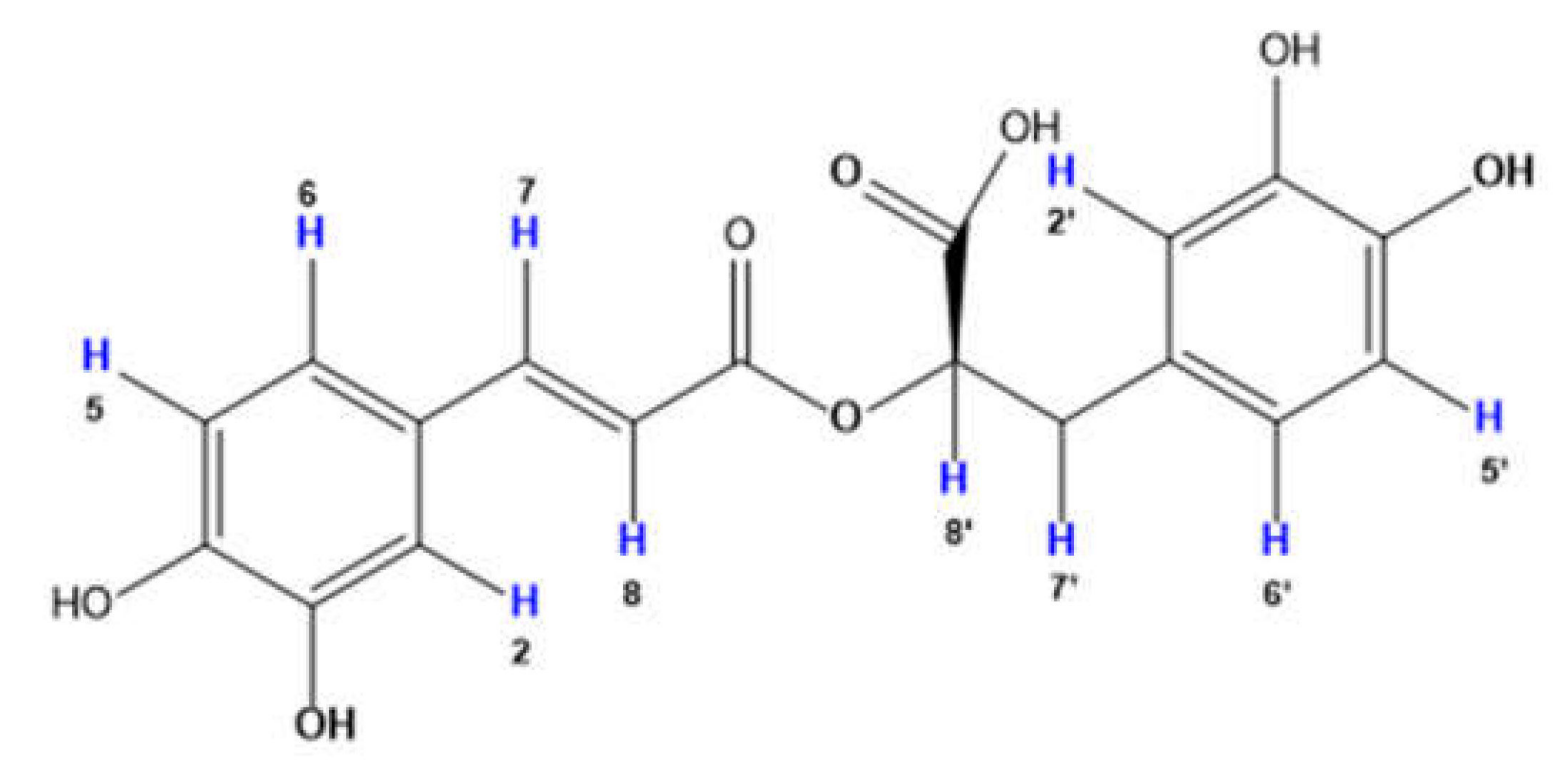
Chemical structure of rosmarinic acid (RA).

Rosmarinic acid (RA) is a polyphenolic compound composed by an ester of caffeic acid and a 3,4-dihydroxyphenyllactic acid (Scheme 1). It is a natural and bioactive molecule found in several plants (Lamiaceae species) generally used as culinary herbs (rosemary, lemon balm, peppermint, and sage).

RA is widely investigated for its biological properties and protective efficacies, such as anti-microbial, immunomodulatory, analgesic and anti-inflammatory, anti-cancer, antioxidant, antidiabetic, neuroprotective, and anti-Alzheimer’s activities [2,3,4,5,6,7,8,9,10,11,12]. The first investigations on the antioxidant effects of RA date to almost 30 years ago [13,14]. After that, the scientific community started to seriously consider RA as a potent nutraceutical and pharmaceutical compound. Over the last ten years, more than 1000 research papers have appeared. In particular, the cellular neuroprotective effects exhibited by RA have made this antioxidant compound a good candidate for treating neurotoxicity and oxidative stress [8,15,16,17,18].

Alzheimer’s disease (AD) is a neurodegenerative disorder related to aging that leads to progressive memory loss and impaired cognitive functions. AD is one of the most severe diseases next to cancer and heart pathologies [19].

On 19th September 2019, the World Health Organization (WHO) announced that (i) around 50 million people have dementia, (ii) 10 million new cases appear every year, and (iii) Alzheimer’s disease is the most widespread form of dementia contributing to 60–70% of cases. Since no treatments are available so far, it is very likely that these numbers will increase to very critical levels in the future due to life expectancy increase. In addition, in December 2017, WHO published the global action plan on the public health response to dementia 2017–2025 [20]. This document describes seven action areas necessary to improve the health and well-being of people affected by dementia. In particular, action areas 4 (dementia diagnosis, treatment, care) and 7 (dementia research and innovations) require the efforts of all the scientific community. Chemists, biologists, physicians, and mathematicians from all over the world have been called to find a cure for dementia and to understand the molecular and cellular pathways associated with neurodegeneration.

The scientific research, to date, has provided the evidence that AD has a multifactorial etiology: several factors of different nature, apparently not directly connected to each other, contribute to triggering the degenerative process of the brain [21,22,23].

Although the causes and the cellular pathways leading to AD pathogenesis are not fully understood, the amyloid cascade hypothesis indicates the accumulation of amyloid β (Aβ) oligomers and fibrils as the main hallmark of AD. Aβ aggregates are very neurotoxic and cause severe damage to neurons; they are responsible for various phenomena, such as oxidation, lipid peroxidation, inflammation, disturbance of cell functions, apoptosis, and neurofibrillary tangles [24,25,26,27,28,29]. For these reasons, most AD therapeutic strategies are mainly centered on Aβ targeting, limiting the possibility to exploit new, potential AD treatments based on the implication of diverse mechanisms of AD etiology. In fact, even if Aβ has a central role in the disease, it cannot be considered as a unique AD causal factor [30,31].

Moreover, transition metal ions, such as copper and zinc, are implicated in the normal neuronal activity. Sophisticated regulatory mechanisms in the brain control these Cu and Zn levels inside the cell and at the synapse interphase. Compelling evidence indicates that both Cu and Zn are dysregulated in AD leading to dramatic impairment of the brain functions [32,33,34,35,36,37,38,39].

Both tau and Aβ proteins are able to bind copper ions [40,41,42,43,44,45,46,47,48,49,50,51]. This interaction has a strong impact on two key events of AD: (i) it influences the aggregation pathways of Aβ and (ii) it leads to the formation of catalytic generation of reactive oxygen species (ROSs). For these reasons, over the last years, many researchers have proposed copper targeting as a possible therapeutic approach to fighting AD. Several investigations have shown the ability of numerous ligands to target copper and to interfere with and recognize Aβ molecules [47,52,53,54,55,56]. In addition to synthetic ligands, multi-target compounds containing natural scaffolds derived from fungi and plant metabolites exhibited valuable biological activity [57].

On these bases, we decided to investigate RA’s ability to interfere with Cu(II)-Aβ association. To address this issue, we first focused on the molecular characterization of the RA–Cu(II) complex by means of UV–VIS, EPR, NMR, and MS techniques. In addition, we evaluated the biological activity of RA and the RA–Cu(II) complex by measuring their cytotoxicity and mutagenicity in vitro. Finally, we analyzed the ternary systems containing Cu(II), RA and Aβ. Our data strongly support the existence of mixed metal complexes, where RA and Aβ are contemporaneously bound to the cupric ion. This association might justify the already known anti-aggregating properties of RA against Aβ [58,59,60,61,62,63] and its ability to attenuate Aβ-induced cellular ROS generation [64,65].

## 2. Materials and Methods

*Materials*—RA and Cu(II) salts (Cu(NO3)_2_ and CuSO_4_ were purchased from Merck (Germany). Aβ16, amyloid β (1–16) and Aβ28, amyloid β (1–28) were purchased from GenScript Biotech Corporation. Aβ16 and Aβ28 peptides are largely used to avoid all the critical handling problems related to the full-length Aβ peptide; they are good models for investigating the Aβ–Cu(II) interaction [41,42,43,44,47]. Dulbecco’s modified Eagle’s medium (DMEM), trypsin solution, and all the solvents used for cell culture were purchased from Lonza (Switzerland). Mouse immortalized fibroblasts NIH3T3 were purchased from American Type Culture Collection (USA). The mutagenicity assay was supplied by Biologik s.r.l. (Trieste, Italy).

*UV–VIS Measurements*—The analyzed samples were prepared from RA and Aβ16 stock solutions in DMSO 0.05 M and diluted with phosphate buffer (1.8 × 10^−2^ M pH 7.4) or distilled water to have a final concentration of 5.0 × 10^−5^ M. Copper titration was done by using stock solution of Cu(NO_3_)_2_ or CuSO_4_ in water. When necessary, pH was adjusted to the desired value by adding NaOH or HCl. The absorption spectra were recorded on a Perkin Elmer Lambda 900 UV/VIS/NIR spectrophotometer.

*EPR Spectroscopy*—The EPR spectra were recorded in liquid nitrogen on a Bruker ELEXSYS E500 CW-EPR spectrometer at X-band frequency (9.5 GHz) and equipped with ER 036TM NMR Teslameter and E41 FC frequency counter. We used 1.0 × 10^−3^ M Cu(II) concentration and 2:1 and 1:1 ligand:Cu(II) molar ratios. Ethylene glycol (20%) was used as a cryoprotectant for EPR measurements in water solution. The EPR parameters were obtained by simulating the experimental spectra with the WIN-EPR SIMFONIA software, version 1.2 (Bruker).

*MS Spectrometry*—High-resolution mass spectra were recorded by using BrukerQ-FTMS spectrometer (Bruker Daltonik, Bremen, Germany) equipped with an Apollo II electrospray ionization source with an ion funnel. Both positive and negative ion modes’ spectra were recorded with the following parameters: scan range, m/z 150–2000; dry gas, nitrogen; temperature, 170 °C; ion energy, 5 eV; transfer time, 120 ps. Capillary voltage was optimized to 4500 V in order to have the best signal to noise (S/N) ratio. Small voltage changes (±500 V) did not significantly alter the optimized spectra. Cu(II) complexes (2:1 and 1:1 ligand:Cu(II) molar ratios respectively, [ligand]_tot_ = 1.0 × 10^−4^ M) were dissolved in a 1:1 MeOH/H_2_O solution at pH 7. The flow rate for sample injection was 3 μL/min. Before each experiment, the instrument was calibrated externally with the Tunemix mixture. Data were processed by using the Compass DataAnalysis 4.0 program (Bruker Daltonic).

*NMR Spectroscopy*—RA solutions were prepared from RA stock solution in DMSO (0.05 M) and diluted with phosphate buffer in D_2_O (1.8 × 10^−2^ M pH 7.4) to have a final concentration of 5 × 10^−4^ M (^1^H NMR experiments) and 5 × 10^−3^ M (^13^C NMR experiments). The desired concentrations of metal ions were achieved by adding small aliquots of Cu(NO_3_)_2_ stock solutions. TSP-d_4_, 3-trimethylsilyl-[2,2,3,3-d_4_] propionate sodium salt was used as the internal reference standard. NMR measurements were performed at 14.1 T with a Bruker Avance 600 MHz spectrometer at controlled temperatures (±0.2 K) using an SEI (sensitive enhancement) probe. The excitation sculpting method was applied for water suppression [66]. A typical ^1^H NMR spectrum required 8 transients, 9 μs 90° pulse and 3.0 s recycling delay. Two-dimensional (2D) NMR ^1^H-^1^H TOCSY and NOESY standard experiments were performed for proton assignment. ^13^C NMR spectra required 8K transients, 15 μs 90° pulse and 5.0 s recycling delay. Carbon resonance assignment was obtained through ^1^H−^13^C HMBC and ^1^H−^13^C HSQC standard 2D sequences. The obtained ^1^H and ^13^C assignments were in agreement with the ones previously reported [67]. Spectra processing was performed using the Bruker TopSpin3.2 software. Proton spin–lattice relaxation rates (R_1_) were determined with inversion recovery (IR) pulse sequences both for the free peptide (R_1f_) and in the presence of the metal ion (R_1obs_). The obtained paramagnetic relaxation enhancements, R_1p_ were calculated by using the following equation:

R_1p_ = R_1obs_ − p_f_R_1f_ (1)

The paramagnetic contributions, R_1p_, are defined as [68,69]:

R_1p_ = p_b_/R_1b_^−1^ + k_off_^−1^ (2)

where k_off_ is the kinetic off rate of ligand molecules in the metal coordination sphere, p_f_ and p_b_ are the fraction of free and bound peptides, R_1obs_ and R_1f_ are the spin–lattice relaxation rates of apo and metal-bound samples and R_1b_ is the rate of ligand nuclei in the metal coordination sphere.

*Cell cultures and cytotoxicity assay*—The in vitro cytotoxicity of the test samples was evaluated by direct contact test, as proposed by ISO 10995-5, Biological evaluation of medical devices—Part 5: Tests for cytotoxicity: in vitro methods [70]. The evaluation of in vitro cytotoxicity does not depend on the final use for which the product is intended, and the document ISO 10995-5:2009 recommends many cell lines from American Type Collection. Among them, to test RA, Cu(II), and RA–Cu(II) cytotoxicity, NIH3T3 mouse fibroblasts were chosen.

*Evaluation of NIH3T3 viability*—Cells were cultured in complete DMEM supplemented with 10% fetal calf serum (FCS), 1% L-glutamine–penicillin–streptomycin solution, and 1% MEM non-essential amino acid solution, and maintained at 37 °C in a humidified atmosphere containing 5% CO_2_. Once the culture media reached confluence, fibroblasts were washed with PBS, detached with trypsin–EDTA solution and then centrifuged at 1000 rpm for 5 min. The pellet was then suspended in complete DMEM with a dilution of 1:15. Cells (1.5 × 10^4^) were seeded in each well of a 24-well round multi-dish and incubated at 37 °C in an atmosphere of 5% CO_2_. After 24 h of culture, when about 50% of the confluence was reached, the culture medium was discharged and the test compounds, properly diluted in completed medium, were added to each well. The stock solution of RA was prepared in Et-OH 60% v/v and the stock solution of Cu(II) in distilled water. The following concentrations of both RA and Cu(II) were tested: 10, 15, 30, 60, and 90 µM. The concentration values of RA–Cu(II) complex tested were 10 µM RA–10 µM Cu(II), 15 µM RA–10 µM Cu(II), 30 µM RA–10 µM Cu(II), 60 µM RA–10 µM Cu(II), and 90 µM RA–10 µM Cu(II). Each experiment was repeated three times and all samples were set up in six replicates. Complete medium was used as negative control. After 24 h of incubation, cell viability was evaluated by neutral red uptake following the procedure previously described [71].

*Mutagenicity assay: Ames test*—The mutagenicity assay was performed by using TA100 and TA98 strains of *Salmonella typhimurium*, with and without metabolic activation, i.e., S9 fraction. Approximately 10^7^ bacteria were exposed to 6 increasing concentrations of each test sample, as well as to both positive and negative control, for 90 min in a medium containing sufficient histidine to support approximately two cell divisions. At the end of incubation, the exposure cultures were diluted in a pH indicator medium lacking histidine, and aliquoted into 48 wells of a 384-well plate. Within two days, cells that had undergone the reversion to His grew into colonies. Metabolism by the bacterial colonies reduced the pH of the medium, changing the color of the medium. This color change can be detected visually. The number of wells containing revertant colonies were counted for each dose and compared to a zero-dose control. Each dose was tested in six replicates. The test was performed both with and without an S9 fraction. The following concentrations of both RA and Cu(II) were tested: 10, 15, 30, 60, 90, and 300 µM. The concentration values of RA–Cu(II) complex tested were: 10 µM RA–10 µM Cu(II), 15 µM RA10–µM Cu(II), 30 µM RA–10 µM Cu(II), 60 µM RA–10 µM Cu(II), 90 µM RA–10 µM Cu(II), and 300 µM RA–300 µM Cu(II).

*Statistical analysis*—Multiple comparison was performed by one-way ANOVA, and individual differences were tested by Fisher’s test after the demonstration of significant intergroup differences by ANOVA. Differences with *p* < 0.05 were considered significant.

## 3. Results

### 3.1. Spectroscopic Characterization of RA–Cu(II) Complex

The interaction between copper(II) ion and RA was evaluated by combining several experimental techniques, such as NMR, UV–VIS, EPR, and MS methods.

Electrospray ionization mass spectrometry (ESI-MS) gave evidence of 2:1 ligand:metal stoichiometry at pH 7 for RA–Cu(II) system (Figure S1). ESI-MS peak assignments were obtained by comparing the precise calculated and experimental m/z values and their isotopic patterns. M/z values at 359.08, 719.16, and 1079.23 correspond to [L]^−^, [L_2_]^−^, and [L_3_]^−^ free ligands, respectively (the sodium adducts of the free ligand are also present (m/z 741.14 for [L_2_+Na]^−^). For the RA–Cu(II) adduct, the m/z 779.10 value is consistent with the existence of bis-complex [CuL_2_]^−^. MS spectra also reveal the presence of a mononuclear [CuL]^−^ complex at m/z 417.04. The experimental isotopic pattern of the ligand and copper complexes is in perfect agreement with the simulated one (Figure S1). The similar set of signals for RA–Cu(II) system were identified on MS spectra recorded in the positive ion mode (additional sodium and water adducts were observed) (Figure S1B). Similar results were obtained by recording MS spectra of the RA–Cu(II) system at a 1:1 molar ratio, (data not shown).

The collected EPR spectra indicate that RA forms stable metal adducts (Figure S2). The spectra were simulated, assuming a spin system with S = 1/2, I = 3/2 for Cu(II), using the g and A tensors. According to Peisach and Blumberg, a correlation plot of g_z_ vs. A_z_ values can be used to identify the types and number of atoms coordinating the copper(II) center [72,73] The spectra acquired at both 2:1 and 1:1 ligand:metal ratios, have almost identical EPR parameters (Figure S2). In particular, their values: g_z_ = 2.32 for both ratios and A_z_ = 154.86 or 154.88 for 1:1 and 2:1 ratio, respectively, clearly indicate Cu(II) binding to oxygen atoms, with the presence of 4O donor set [72,73].

**Figure 1:**
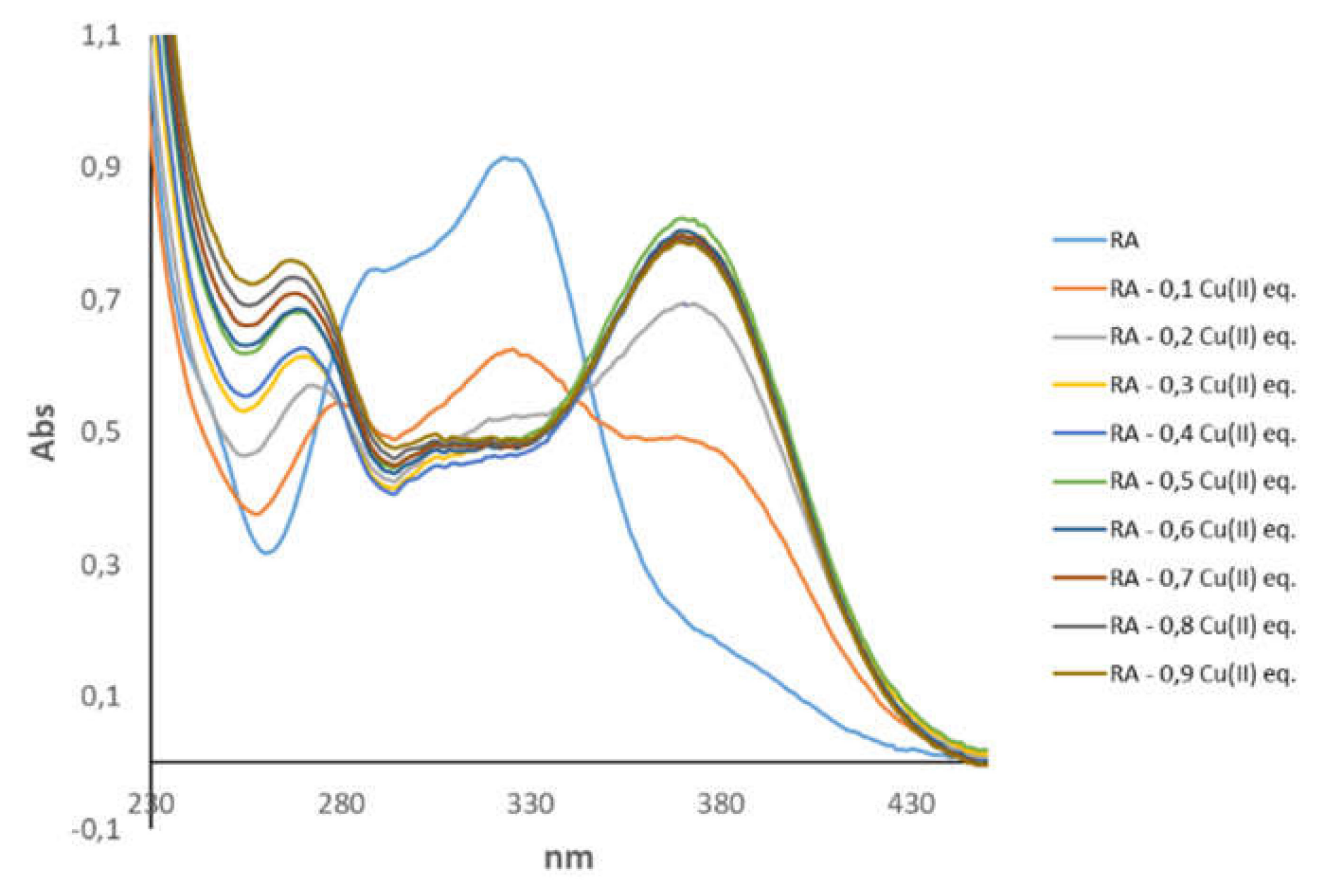
UV–VIS spectra of rosmarinic acid (RA) at pH 7.4 without and with increasing Cu(II) concentrations. [RA] = 5.0 × 10^−5^ M; [phosphate buffer] = 1.8 × 10^−2^ M. Comma is used to indicate decimal point in the picture.

The binding of Cu(II) ions was also monitored by UV–VIS analysis, where RA was titrated with increasing amounts of the paramagnetic ions. The metal-induced changes were evaluated by looking at electronic transitions originated either by Cu(II) ions (d–d transitions) or RA chromophore groups. As expected, d–d transitions have a much lower intensity. In both cases, we observed that UV–VIS absorption bands reached intensity plateau in the presence of 0.3–0.4 Cu(II) equivalents (Figure 1 and Figure S3), thus supporting the formation of bis-complex, where Cu(II) coordinates two RA molecules.

At pH 7.0, RA is characterized by a UV–VIS spectrum with two intense bands at 287 and 327 nm, in agreement with previous data [74]. These bands are strongly dependent on pH values and correlate with specific deprotonated or protonated RA species [74]. Binding of Cu(II) results in a substantial change of the electronic properties of RA and gives a spectrum with two maximum absorptions at 262 and 370 nm. The shape of the UV–VIS spectrum of the complex strongly resembles the one of RA at basic pH (Figure S4), where OH deprotonation gives specific UV–VIS features [74]. The close similarity between the UV–VIS spectrum of RA–Cu(II) complex and RA at basic pH (Figure S4) strongly indicates Cu(II) interaction with deprotonated OH groups.

### 3.2. Molecular Structure of RA–Cu(II) Complex

**Figure 2:**
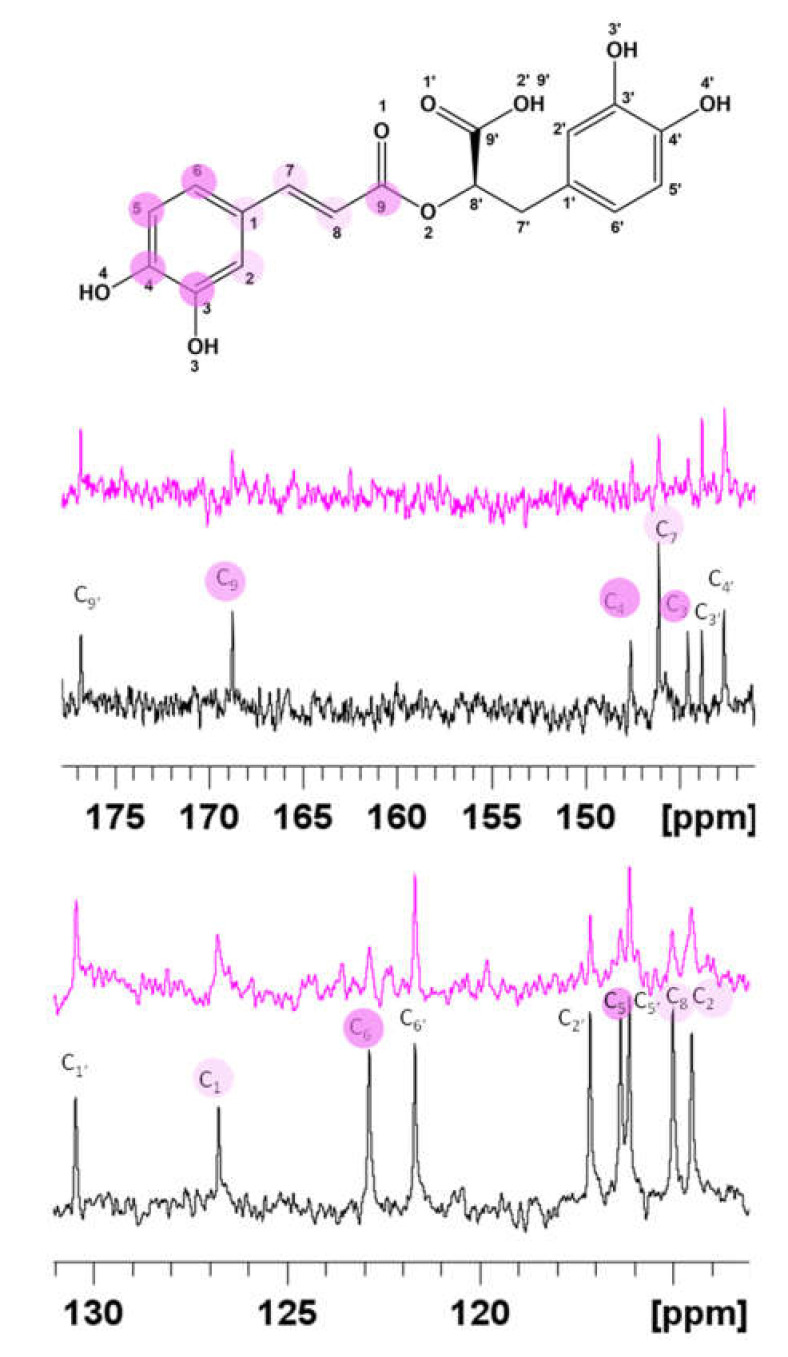
Superimposition of ^13^C NMR spectra in absence (apo, black line) and in presence of 0.02 Cu(II) eqs. (magenta line). [RA] = 5.0 × 10^−3^ M; [Cu(II)] = 1.0 × 10^−4^ M [phosphate buffer] = 1.8 × 10^−2^ M.

A detailed structural characterization of the RA–Cu(II) complex was obtained by performing a full NMR analysis. By comparing 1D and 2D spectra of RA in the absence and presence of copper(II), we observed a selective line broadening of NMR resonances (Figure S5 and Figure 2). Such behavior is in agreement with previous NMR studies of Cu(II)–ligand interactions, and it is consistent with the dipolar interaction between the Cu(II)–unpaired electron and RA nuclei close to the paramagnetic center [75,76,77,78].

Copper’s effects on ^1^H and ^13^C line broadening were diverse according to the difference between the proton and carbon gyromagnetic ratio [75]. As reported in Figure 2, the resonances belonging to the caffeic acid portion (magenta circles) are the most affected. No significant variations are evident on the carboxylic group. Such findings strongly point out that the two hydroxyl groups at positions 3 and 4 are bound to the copper ions and exclude the involvement of the carboxylate.

**Figure 3:**
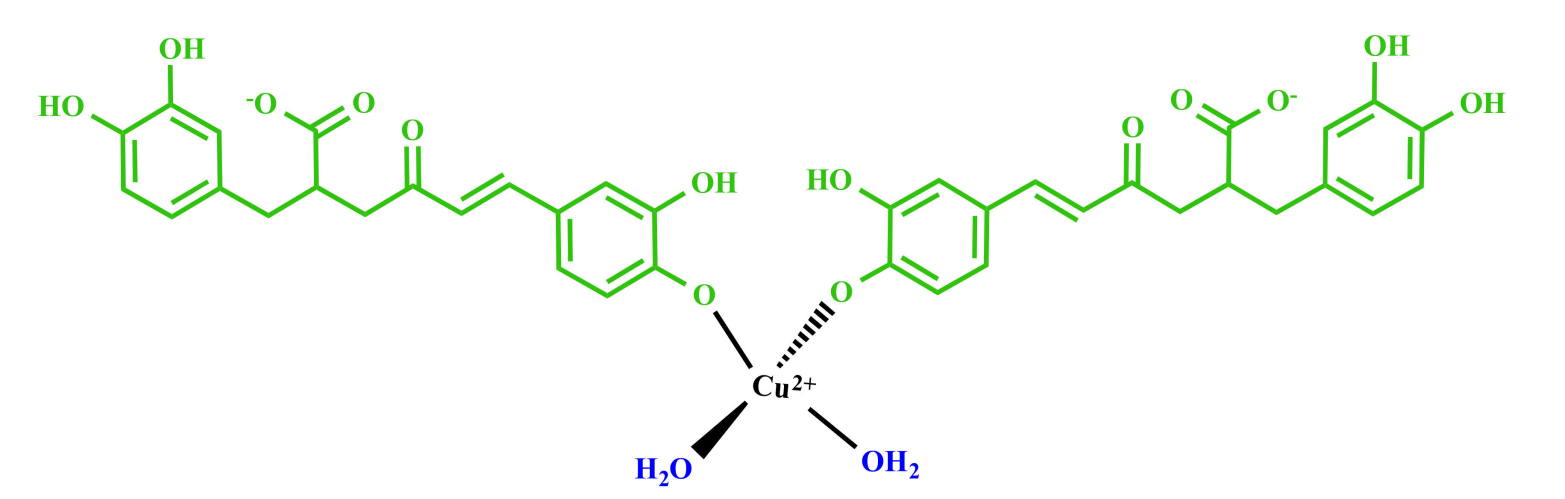
Proposed chemical structures of RA–Cu(II) complex.

The effects of Cu(II) on ^1^H resonances were also determined by measuring the longitudinal proton relaxation enhancements (R_1p_) induced by the cupric ion. Such values provide information on the nuclei closer to the paramagnetic center, and they were calculated in presence of either 0.01 or 0.02 Cu(II) eqs. (Figure S6). The largest values were measured for H2 and H5 protons, which are the closest ones to the OH groups, thus confirming their participation as binding donor atoms in the copper(II) coordination sphere. In addition to H2 and H5, other NMR signals (H6, H7, and H8) show significant paramagnetic effects (R_1p_) consistent with their proximity to the metal center. Finally, we measured very small values for the protons (H2′, H5′, and H5′) belonging to the other aromatic ring. All the collected data are consistent with Cu(II) binding to 2 RA molecules with a 4 oxygen donor set. NMR analysis identified deprotonated OH groups of the caffeic acid portions as the metal coordination site. According to that, we have proposed the following structure (Figure 3): Cu(II) deprotonates and coordinates to the most acidic phenolate oxygens of the caffeic portion. Two water molecules complete the metal coordination sphere.

### 3.3. In Vitro Cytotoxicity and Mutagenicity of RA and RA–Cu(II) Complex

**Figure 4:**
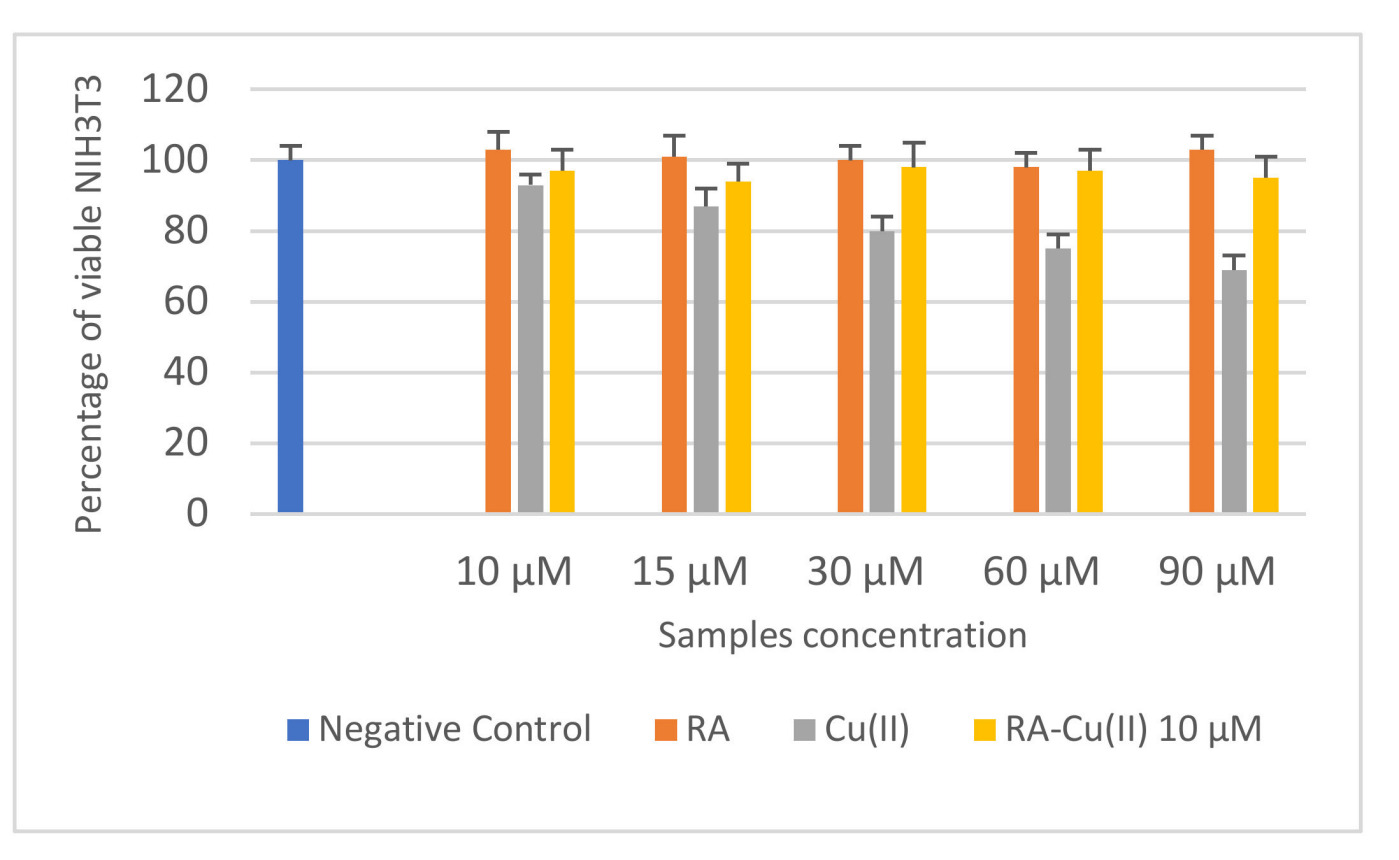
Percentage of viable NIH3T3 after 24 h of contact with different concentrations of RA, Cu(II), and RA–Cu(II) as determined by the neutral red uptake. Data are mean SD of three experiments run in six replicates. Values are statistically different versus negative control (complete medium), *p* < 0.05.

Non-confluent adhered NIH3T3 were incubated with different concentrations of RA, Cu(II), and RA–Cu(II) complex. Cell viability was evaluated after 24 h, and the results are reported in Figure 4. RA did not reduce the percentage of cell viability in comparison to negative control for all the concentration values tested, demonstrating noninterference with the cell cycle. On the contrary, cells in contact with Cu(II) decreased, in comparison to those in contact with the negative control, by increasing metal ion concentration, demonstrating the cytotoxic effect of Cu(II) at concentrations >10 µM. The complex between Cu(II) 10 µM and increasing concentrations of RA (10–90 µM) was not toxic toward fibroblasts. Cell density was in fact the same as the that of the negative control.

As a further biological characterization, we evaluated the mutagenicity in *S. typhimurium* strains TA98 and TA100 (Figures S7 and S8). The assay was performed both in the presence and absence of rat liver S9 fraction, which allows a more in-depth investigation on the potential mutagenicity risks derived from the test compound metabolites. Our data (Figures S7a,b and S8a,b), indicate that RA did not show any mutagenic effects at all the concentrations tested on TA98 and TA100 strains, both with and without the S9 fraction. On the contrary, (Figures S7c,d and S8c,d), for both TA98 and TA100 strains, Cu(II) demonstrated a mutagenic effect that increased by increasing the tested concentration of metal ion both with and without S9 fraction. Only Cu(II) concentrations of 10 and 15 µM had no mutagenic effect toward TA98 and TA100, and this behavior was independent of the presence of the S9 fraction.

The presence of RA was not able to reduce Cu(II) mutagenicity as reported in Figures S7e,f and S8e,f; the complex was shown, in fact, to be mutagenic in a dose-dependent manner for concentrations ≥30 µM. Therefore, although the presence of RA was able to reduce Cu(II) cell toxicity, it had a concentration-dependent effect toward metal ion mutagenicity.

### 3.4. Spectroscopic Characterization of RA–Cu(II)–Aβ System

Amyloid β (Aβ) is able to bind Cu(II) and Cu(I) ions by forming stable metal complexes, as recently reviewed [41,42,43,44]. In addition, RA is able to interact with Aβ and to induce neuroprotective effects [60,63]. On that basis, we decided to understand the behavior of the Aβ–Cu(II) complex with the simultaneous presence of RA, which might sequester Cu(II) from Aβ and interact with it, as found for other natural compounds able to mediate and affect the pathways leading to the formation of metal-induced toxic oligomers [57].

All the data were collected by using the first N-terminal 16 amino acids Aβ (Aβ16), which is considered as a good model for studying Cu(II) binding [41,42,43,44,51].

**Figure 5:**
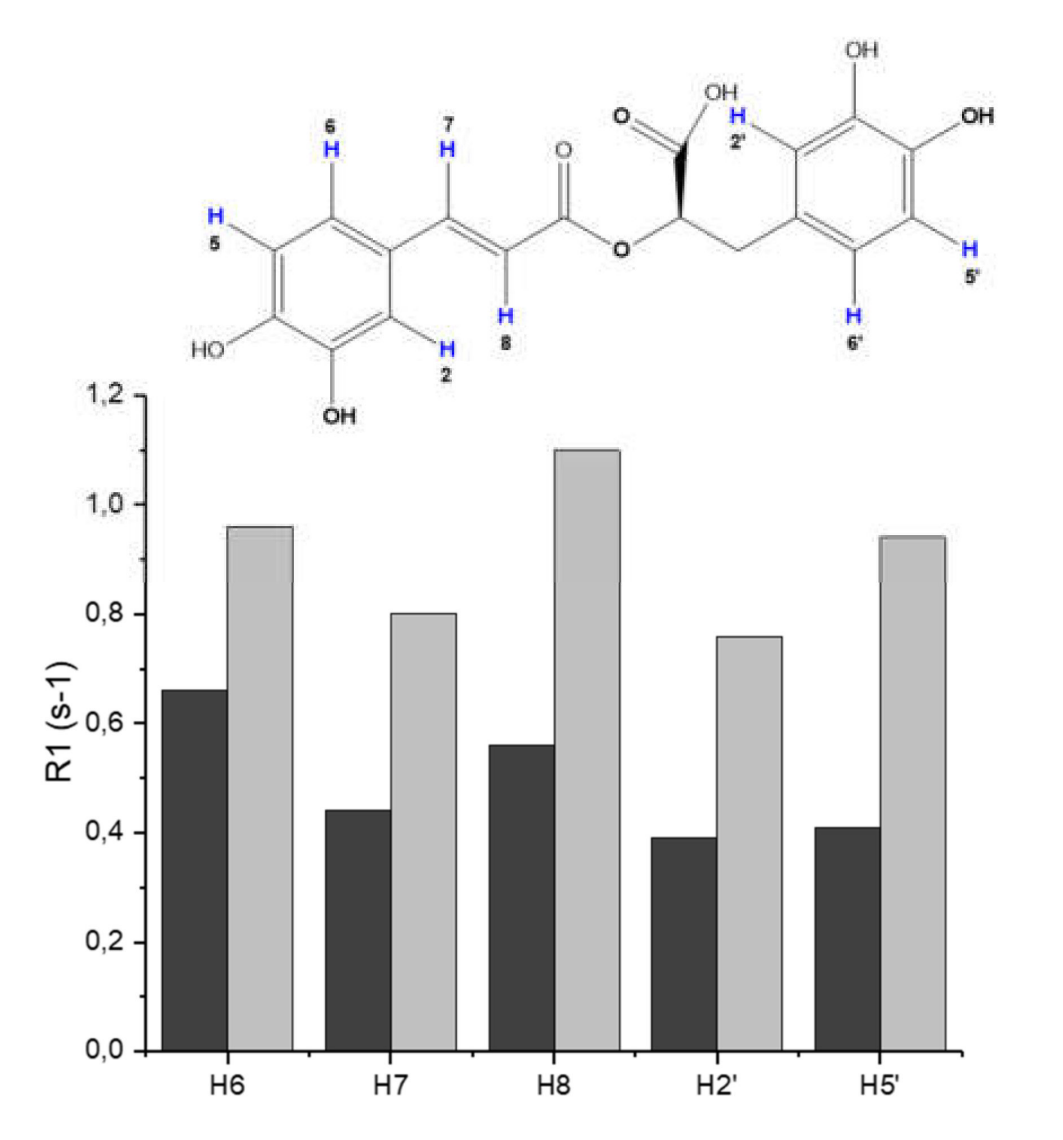
R1 values of RA protons measured in solutions without (dark grey) and with amyloid β (Aβ) (light grey). [RA] = [Aβ] = 5.0 × 10^−4^ M; [phosphate buffer] = 1.8 × 10^−2^ M.

Upon addition of Aβ16 to RA solutions, we did not observe any detectable chemical shift variations in RA signals. On the other hand, we found a significant increase of the longitudinal relaxation rates of RA protons (Figure 5). Such data are consistent with a slowing down of the molecular tumbling of RA in the presence of Aβ16 and strongly indicate a week association between RA and Aβ16. Our data are in agreement with previous NMR findings showing RA interaction with Aβ oligomer by saturation transfer difference (STD) and transferred NOESY (trNOESY) experiments [79]. Airoldi et al. suggested a possible role played by the conjugated double bond in the recognition and binding processes. Interestingly, our data show the large increase of H7 and H8 relaxation rates, whose values are twice as big.

For the following step, we decided to verify the behavior of Cu(II) in samples simultaneously containing RA and Aβ16. Our intent was to understand how Cu(II) distributes between the two ligands and to monitor the possible existence of a ternary interaction where Cu(II) binds both RA and Aβ16 in a unique complex, thus favoring an RA–Aβ interaction.

**Figure 6:**
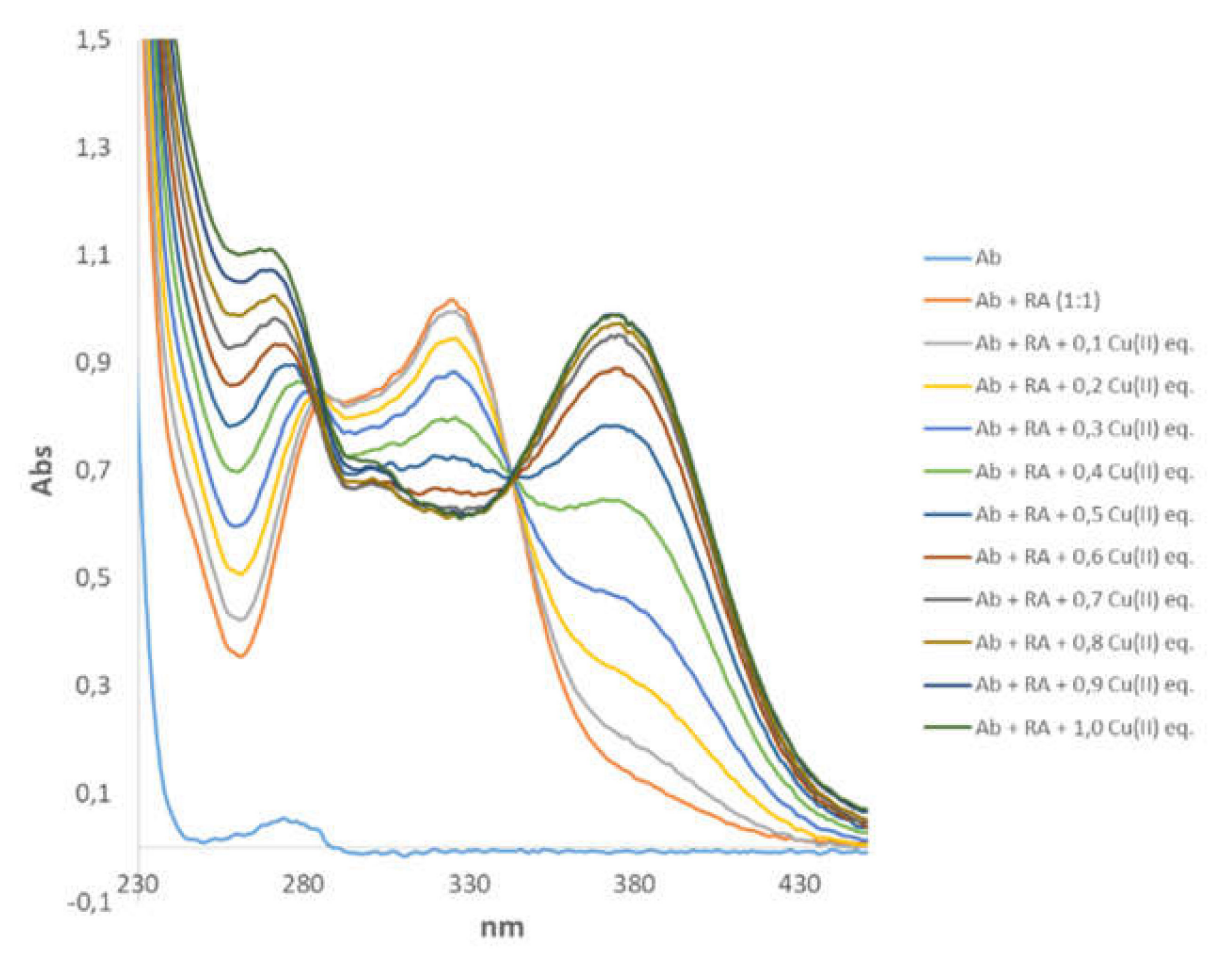
UV–VIS spectra of RA and Aβ16 at pH 7.4 without and with increasing Cu(II) concentrations. [RA] = [Aβ] 5.0 × 10^−5^ M; [phosphate buffer] = 1.8 × 10^−2^ M. Comma is used to indicate decimal point in the picture.

To address this issue, we applied NMR, UV–VIS, and EPR spectroscopies. As is easily evident in Figure 6, the contemporaneous presence of RA and Aβ16 caused variations on the UV–VIS spectrum, where the main difference consists in the metal amount necessary to reach the intensity plateau of the UV–VIS absorption bands, which passed from 0.4 to 0.9 Cu(II) eqs for RA–Cu(II) and RA–Aβ16–Cu(II) systems, respectively. On the other hand, the spectral features of RA were still conserved, strongly indicating that (i) RA is still bound to Cu(II) and (ii) Cu(II) still binds to the more acid phenolate groups.

Similarly to the UV–VIS data, we also observed changes of EPR spectra recorded for RA–Cu(II)–Aβ16 (0.5:1:1) and RA–Cu(II)–Aβ28 (0.5:1:1) systems, whose features are completely different from those of the RA–Cu(II) complex (Figure S9). The g_z_ and A_z_ values for RA–Cu(II)–Aβ16 (g_z_ = 2.265, A_z_ = 172.8) and RA–Cu(II)–Aβ28 (g_z_ = 2.275, A_z_ = 164.32) indicate the involvement of two nitrogen atoms in copper coordination. Similar sets of EPR parameters were obtained for 1:1:1 (RA:Cu(II):Aβ) molar ratio (data not shown). The obtained EPR parameters are in good agreement with the Peisach–Blumberg plot for 2N2O donors [72], suggesting that RA is still bound to Cu(II) by OH groups. The coordination mode {2N2O} was verified by comparing our data with others presented in the literature containing the same donor sets [80,81]. In addition, the comparison between our EPR parameters and the ones of Aβ16–Cu(II) and Aβ28–Cu(II) complexes at the same pH [46,82,83,84], points out a diversity in the metal binding modes. The copper donor set changes from {3N1O} to {2N2O} in Aβ and Aβ–RA systems, respectively.

**Figure 7:**
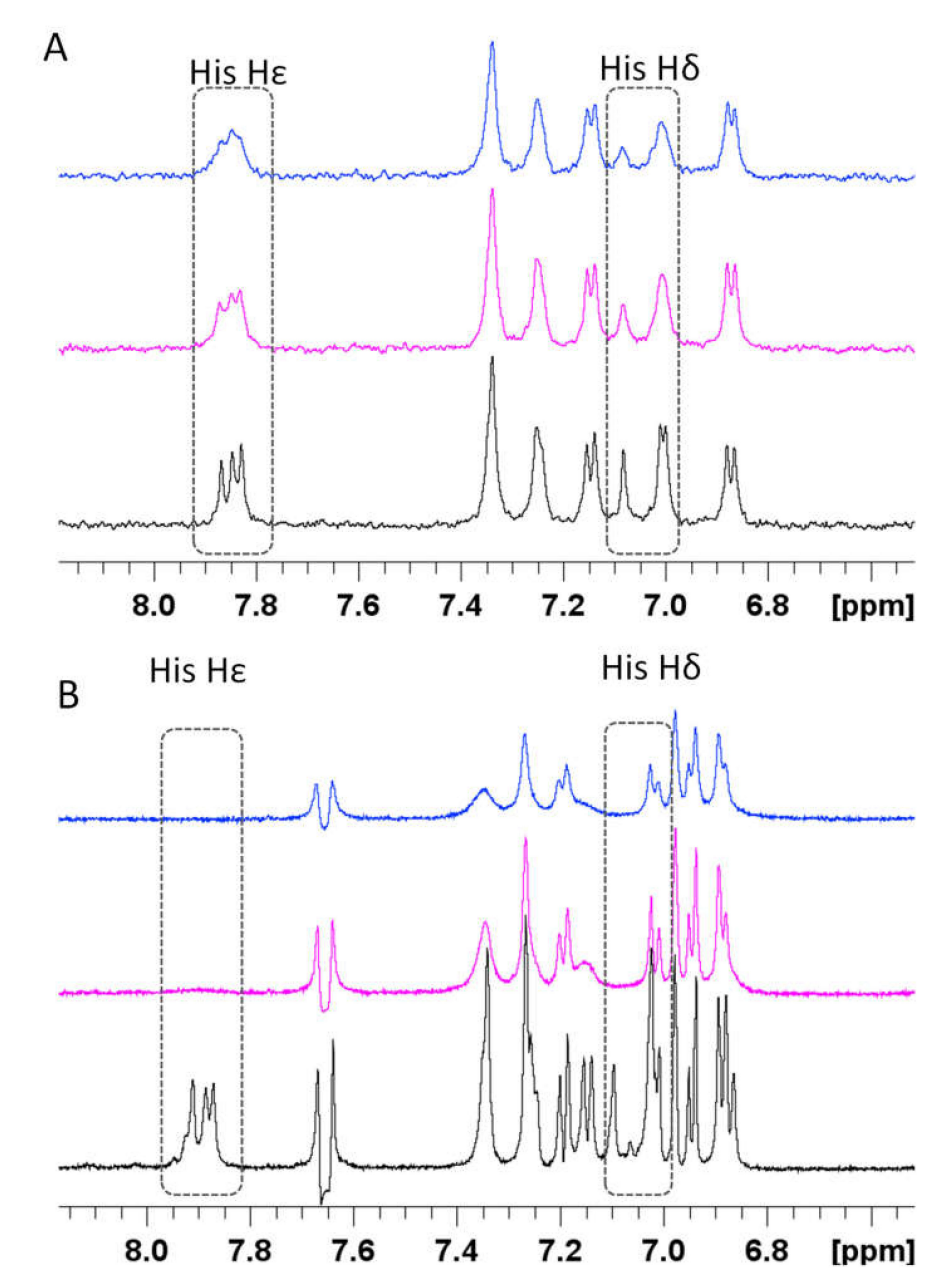
Superimposition of ^1^H NMR spectra in absence (black line) and in presence of 0.02 (magenta) and 0.04 (blue) Cu(II) eqs. (**A**) Aβ16 and (**B**) RA–Aβ16. [RA] = [Aβ] 5.0 × 10^−4^ M; [phosphate buffer] = 1.8 × 10^−2^ M.

**Figure 8:**
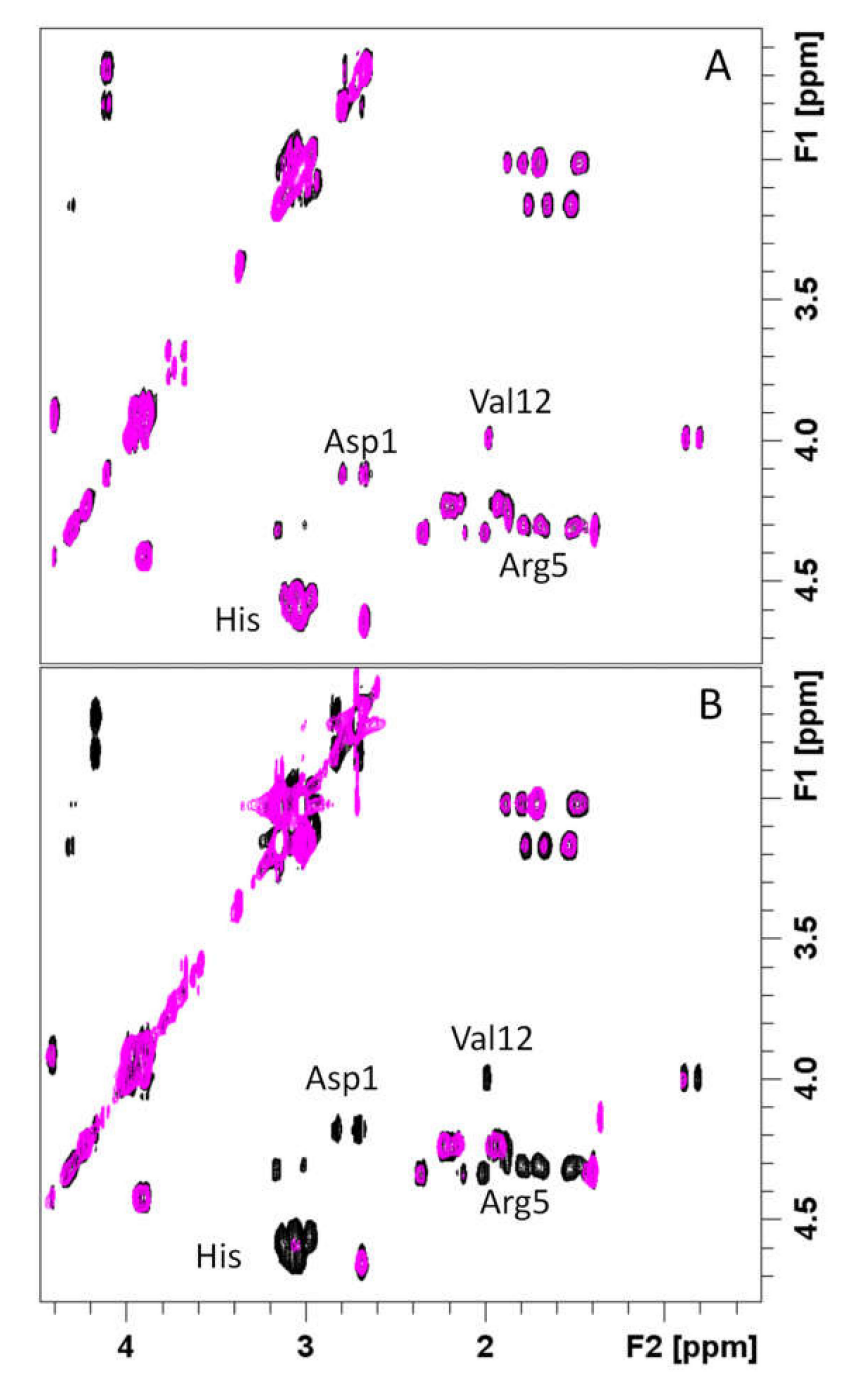
Superimposition of 2D NMR ^1^H-^1^H TOCSY spectra in absence (black line) and in presence of 0.04 (magenta) Cu(II) eqs. (**A**) Aβ16 and (**B**) RA–Aβ16. [RA] = [Aβ] 5.0 × 10^−4^ M; [phosphate buffer] = 1.8 × 10^−2^ M.

NMR investigations on RA–Cu(II)–Aβ16 system showed that Cu(II) addition caused selective line broadening and relaxation rate enhancements on both Aβ16 and RA signals (Figures S10 and S11). In particular, the aromatic and aliphatic protons of the three His (Aβ16) are completely washed out. Similar effects are evident on aliphatic protons of Ala-1, Arg-5, Val-12, and Lys-16 as well. We also measured the paramagnetic relaxation enhancement (R_1p_) on RA aromatic protons. Unfortunately, we could not include all the RA signals but only those that were not overlapping with the ones belonging to Aβ16. The data, reported in Figure S11, indicate the largest variations in H6 protons, suggesting that the caffeic acid portion is still the metal coordinating region, as previously found for RA–Cu(II) complex alone (see below). Moreover, the comparison of R_1p_ values measured in presence of 0.02 Cu(II) eqs., either with or without the simultaneous presence of Aβ16 (Figure S12) shows that, although the trend is maintained, the values measured in presence of Aβ16 are much smaller. On the other hand, the comparison between the line broadenings induced by Cu(II) on Aβ16, either in absence or in presence of RA, shows that the same amount of the paramagnetic ion (0.04 eqs.) causes much more dramatic effects when RA is in solution as well (Figure 7 and Figure 8). This different trend exhibited by Aβ16 NMR signals, according to the simultaneous presence or absence of RA, strongly indicates RA’s ability to interfere with the Aβ–Cu(II) association. Figure 7 shows the effects of Cu(II) addition on His aromatic protons: while slightly affected in the Aβ16–Cu(II) complex, they completely vanished in the RA–Cu(II)–Aβ16 system. Similar behavior is observed by comparing 2D NMR ^1^H-^1^H TOCSY spectra, where copper(II) caused the disappearance of resonances of His, Asp1, Arg-5, and Val-12 in the RA–Cu(II)–Aβ16 system only (Figure 8).

As previously reported, the extent of the paramagnetic line broadening and relaxation enhancements depends either on the metal-bound molar fraction or on the kinetic off rate of the complex [75,76,77,78]. The higher the metal-bound molar fraction is the larger is the paramagnetic effect. The faster the metal complex dissociation the larger the paramagnetic effect. Since the same amount of Cu(II) was added to Aβ16–Cu(II) and Aβ16–Cu(II)–RA, we can exclude the increase of the molar fraction as the cause of the large line broadening. In fact, at the very least, we should observe a decrease of the molar fraction due to the eventual Cu(II) sequestering by RA. As a consequence, the largest Cu(II) effects observed on Aβ16–Cu(II)–RA might be explained by considering the formation of a new complex with different kinetic features. The diverse kinetic off rate is also consistent with the increase of R_1p_ values measured for RA protons in presence of an equimolar amount of Aβ16 (Figure S12).

All these findings strongly support the formation of a new complex where RA and Aβ16 contemporaneously bound to the cupric ion. In particular, NMR, UV–VIS, and EPR data point out that Cu(II) is coordinated to deprotonated oxygen atoms of the caffeic acid portions and two nitrogen donors provided from Aβ16.

**Figure 9:**
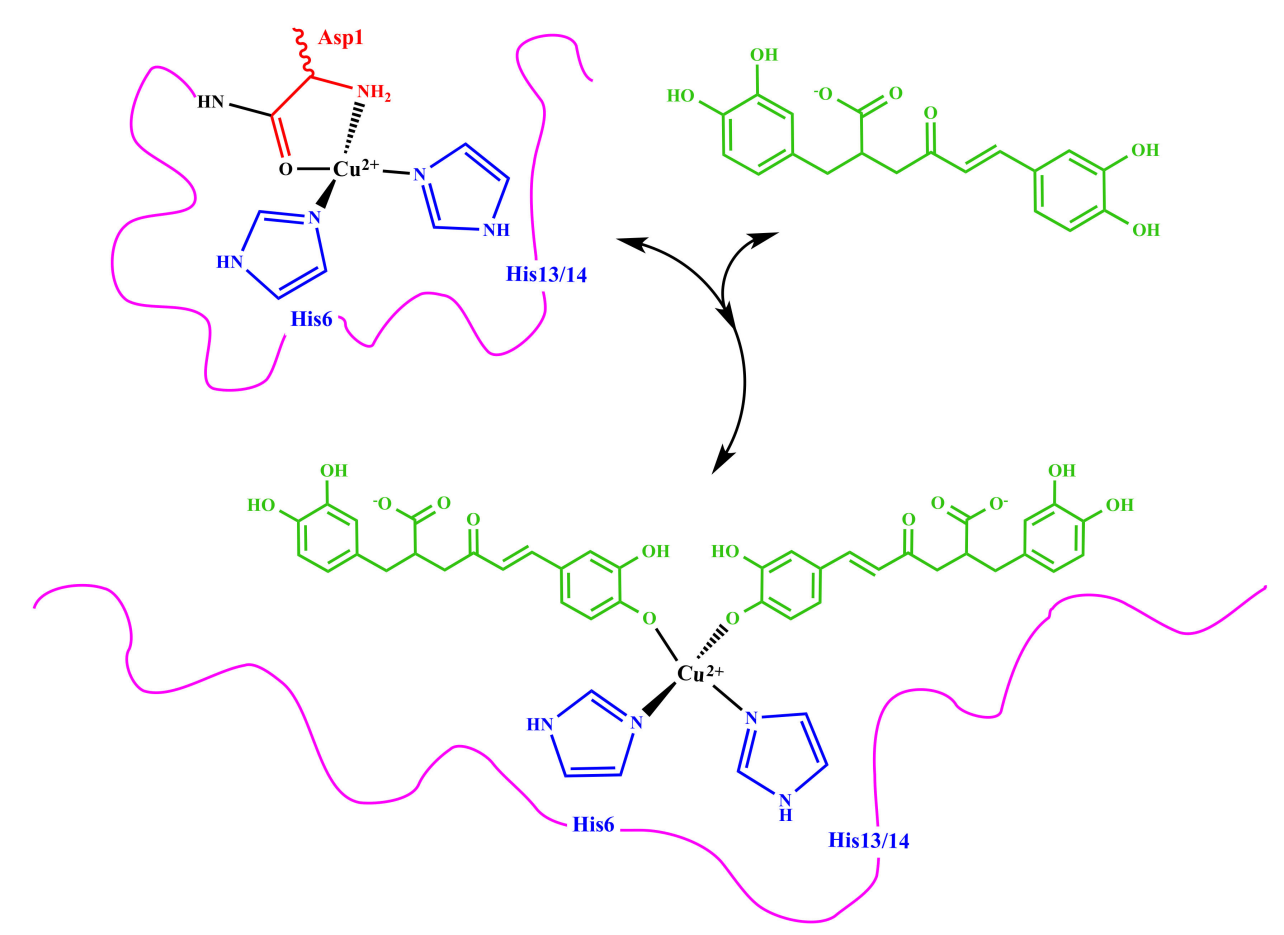
Proposed chemical structures of RA–Aβ–Cu(II) complex.

Our data strongly indicate that RA is able to perturb Cu(II)–Aβ association, leading to a partial rearrangement of the metal coordination sphere as indicated in Figure 9. Once RA is bound to Cu(II) and Aβ, it might exert its antioxidant and anti-aggregating activities, thus preventing the formation of ROSs and oligomeric species.

## 4. Discussion

Our findings strongly indicate that RA is able (i) to bind Cu(II) by forming a bis-complex with 4 oxygen donor atoms and (ii) to mediate the interaction between Aβ and the paramagnetic ion, with the formation of a mixed Cu(II) complex. NMR, UV–VIS, and EPR data gave evidence of tetra-coordinated Cu(II) with 2N2O donor set. Copper-bound oxygen atoms belong to RA (caffeic acid portions), while nitrogen atoms are provided by Aβ (probably His or N-terminus).

It is well accepted that Cu(II) binding to Aβ has very detrimental effects by accelerating Aβ aggregation and ROS formation [41,44,46]. On the other hand, RA is a polyphenolic compound and is able to weakly interact with Aβ and to reduce Aβ-mediated cellular toxicity. The weak RA–Aβ association suggest that RA may act as a pan-assay-interference (PAINS) rather than as a specific and active Aβ ligand. However, in vitro studies indicate that RA is able to inhibit the formation of Aβ fibrils and to destabilize the preformed ones. Moreover, in vivo studies show that RA treatment prevents the development of AD pathology in Tg2576 mice [58,59,60,61], strongly supporting its beneficial role. In addition, RA has also antioxidant properties and it attenuates ROS generation and lipid hydroperoxides caused by Aβ [11,65].

On the basis of these evidences, our data suggest that the biological activity of RA might be strictly correlated to the cellular processes affected by the anomalous and toxic Cu(II)–Aβ interaction, thus exerting a protective role in the cells. In fact, the Aβ–Cu(II) and the mixed RA–Cu(II)–Aβ complexes have different chemical features, such as the metal coordination sphere and the kinetic off rate. This different behavior can have a strong impact on the chemical reactivity of Aβ in terms of aggregation and ROS generation. In addition, our biological analysis performed on RA indicates that it is well tolerated by the cells, which have an almost unperturbed viability in the presence of RA and the RA–Cu(II) complex. RA has no mutagenic effects as well, supporting its relevance in the nutraceutical and pharmacological field.

## Supplementary Materials

The following are available online at https://www.mdpi.com/2075-1729/10/7/118/s1, Figure S1. ESI-MS spectra of Cu(II)–RA; Figure S2. EPR spectra of RA:Cu(II) system; Figure S3. UV–VIS spectra (d–d transitions) of RA at pH 7.4 in presence of increasing Cu(II) concentrations; Figure S4. Comparison between UV–VIS spectra of RA–Cu(II) complex at pH 7.4 and of RA at basic pH. Figure S5. Superimposition of ^1^H NMR spectra in absence and in presence of increasing concentration of Cu(II) ions; Figure S6. R1p values of RA protons measured in solutions with 0.01 Cu(II); Figure S7. Ames test performed on Salmonella typhimurium strain TA98, with and without S9 fraction; Figure S8. Ames test performed on S. typhimurium strain TA100, with and without S9 fraction; Figure S9. EPR spectra of (a) RA–Cu(II), (b) Aβ16–RA–Cu(II), (c) Aβ28–RA–Cu(II) systems; Figure S10. Superimposition of 2D NMR ^1^H-^1^H TOCSY of Aβ16–RA (black contours) and Aβ16–RACu(II); Figure S11. R1p values of RA protons measured in Aβ16–RA solutions with 0.02 Cu(II) eqs.; Figure S12. Comparisons between R1p values of RA protons measured for Aβ16–RA–Cu(II) and RA–Cu(II).

## References

[1] Palamidessi T. (1966). Guarire con le Piante Medicinali.

[2] Wang Y.S., Shen C.Y., Jiang J.G. (2019). Antidepressant Active Ingredients from Herbs and Nutraceuticals Used in TCM: Pharmacological Mechanisms and Prospects for Drug Discovery. Pharmacol. Res..

[3] Anwar F., Abbas A., Mehmood T., Gilani A.H., Rehman U.N. (2019). Mentha: A genus rich in vital nutra-pharmaceuticals—A review. Phytother. Res..

[4] Elufioye T.O., Habtemariam S. (2019). Hepatoprotective effects of rosmarinic acid: Insight into its mechanisms of action. Biomed. Pharmacother..

[5] Fachel F.N.S., Schuh R.S., Veras K.S., Bassani V.L., Koester L.S., Henriques A.T., Braganhol E., Teixeira H.F. (2019). An Overview of the Neuroprotective Potential of Rosmarinic Acid and Its Association with Nanotechnology-Based Delivery Systems: A Novel Approach to Treating Neurodegenerative Disorders. Neurochem. Int..

[6] Swamy M.K., Sinniah U.R. (2018). A Ghasemzadeh Anticancer Potential of Rosmarinic Acid and Its Improved Production Through Biotechnological Interventions and Functional Genomics. Appl. Microbiol. Biotechnol..

[7] Andrade J.M., Faustino C., Garcia C., Ladeiras D., Reis C.P., Rijo P. (2018). *Rosmarinus officinalis* L.: An Update Review of Its Phytochemistry and Biological Activity. Future Sci. OA.

[8] Habtemariam S. (2018). Molecular Pharmacology of Rosmarinic and Salvianolic Acids: Potential Seeds for Alzheimer’s and Vascular Dementia Drugs. Int. J. Mol. Sci..

[9] Alagawany M., El-Hack M.E.A., Farag M.R., Gopi M., Karthik K., Malik Y.S., Dhama K. (2017). Rosmarinic Acid: Modes of Action, Medicinal Values and Health Benefits. Anim. Health Res. Rev..

[10] Bulgakov V.P., Vereshchagina Y.V., Veremeichik G.N. (2018). Anticancer Polyphenols From Cultured Plant Cells: Production and New Bioengineering Strategies. Curr. Med. Chem..

[11] Moore J., Yousef M., Tsiani E. (2016). Anticancer Effects of Rosemary (*Rosmarinus officinalis* L.) Extract and Rosemary Extract Polyphenols. Nutrients.

[12] Shen C.Y., Jiang J.G., Yang L., Wang D.W., Zhu W. (2017). Anti-ageing Active Ingredients from Herbs and Nutraceuticals Used in Traditional Chinese Medicine: Pharmacological Mechanisms and Implications for Drug Discovery. Br. J. Pharmacol..

[13] Lamaison J.L., Petitjean-Freytet C., Carnat A. (1990). Rosmarinic Acid, Total Hydroxycinnamic Derivatives and Antioxidant Activity of Apiaceae, Borraginaceae and Lamiceae Medicinals. Ann. Pharm. Fr..

[14] Lamaison J.L., Petitjean-Freytet C., Carnat A. (1991). Medicinal Lamiaceae with Antioxidant Properties, a Potential Source of Rosmarinic Acid. Pharm. Acta Helv..

[15] Nabavi S.F., Tenore G.C., Daglia M., Tundis R., Loizzo M.R., Mohammad N.S. (2015). The Cellular Protective Effects of Rosmarinic Acid: From Bench to Bedside. Curr. Neurovascular Res..

[16] Kelsey N.A., Wilkins H.M., Linseman D.A. (2010). Nutraceutical Antioxidants as Novel Neuroprotective Agents. Molecules.

[17] Orhan I.E., Senol F.S., Ozturk N., Akaydin G., Sener B. (2012). Profiling of in Vitro Neurobiological Effects and Phenolic Acids of Selected Endemic Salvia Species. Food Chem..

[18] Bigford G.E., del Rossi G. (2014). Supplemental Substances Derived from Foods as Adjunctive Therapeutic Agents for Treatment of Neurodegenerative Diseases and Disorders. Adv. Nutr..

[19] James B.D., Leurgans S.E., Hebert L.E., Scherr P.A., Yaffe K., Bennett D.A. (2014). Contribution of Alzheimer Disease to Mortality in the United States. Neurology.

[20] WHO (2017). Global Action Plan on the Public Health Response to Dementia 2017–2025.

[21] Takeda S. (2019). Progression of Alzheimer’s Disease, Tau Propagation, and Its Modifiable Risk Factors. Neurosci. Res..

[22] Dorszewska J., Prendecki M., Oczkowska A., Dezor M., Kozubski W. (2016). Molecular Basis of Familial and Sporadic Alzheimer’s Disease. Curr. Alzheimer Res..

[23] Rollo J.L., Banihashemi N., Vafaee F., Crawford J.W., Kuncic Z., Holsinger R.M. (2016). Unraveling the Mechanistic Complexity of Alzheimer’s Disease Through Systems Biology. Alzheimers Dement..

[24] Penke B., Bogár F., Paragi G., Gera J., Fülöp L. (2019). Key Peptides and Proteins in Alzheimer’s Disease. Curr. Protein Pept. Sci..

[25] Guix F.X. (2020). He Interplay Between Aging-Associated Loss of Protein Homeostasis and Extracellular Vesicles in Neurodegeneration. J. Neurosci. Res..

[26] Taylor J.P., Hardy J., Fischbeck K.H. (2002). Toxic Proteins in Neurodegenerative Disease. Science.

[27] Hardy J., Selkoe D.J. (2002). The Amyloid Hypothesis of Alzheimer’s Disease: Progress and Problems on the Road to Therapeutics. Science.

[28] Karran E., de Strooper B. (2016). The Amyloid Cascade Hypothesis: Are We Poised for Success or Failure?. J. Neurochem..

[29] Chun W., Johnson G.V. (2007). The Role of Tau Phosphorylation and Cleavage in Neuronal Cell Death. Front. Biosci..

[30] Mehta D., Jackson R., Paul G., Shi J., Sabbagh M. (2017). Why do trials for Alzheimer’s disease drugs keep failing? A discontinued drug perspective for 2010–2015. Expert Opin. Investig. Drugs.

[31] Morris G.P., Clark I.A., Vissel B. (2018). Questions concerning the role of amyloid-beta in the definition, aetiology and diagnosis of Alzheimer’s disease. Acta Neuropathol..

[32] Sensi S.L., Granzotto A., Siotto M., Squitti R. (2018). Copper and Zinc Dysregulation in Alzheimer’s Disease. Trends Pharmacol. Sci..

[33] Adlard P.A., Bush A.I. (2018). Metals and Alzheimer’s Disease: How Far Have We Come in the Clinic?. J. Alzheimers Dis..

[34] Mathys Z.K., White A.R. (2017). Copper and Alzheimer’s Disease. Adv. Neurobiol..

[35] Avan A., Hoogenraad T.U. (2015). Zinc and Copper in Alzheimer’s Disease. J. Alzheimers Dis..

[36] Bulcke F., Dringen R., Scheiber I.F. (2017). Neurotoxicity of Copper. Adv. Neurobiol..

[37] Mezzaroba L., Alfieri D.F., Simão A.N.C., Reiche E.M.V. (2019). The Role of Zinc, Copper, Manganese and Iron in Neurodegenerative Diseases. Neurotoxicology.

[38] Gaggelli E., Kozlowski H., Valensin D., Valensin G. (2006). Copper Homeostasis and Neurodegenerative Disorders (Alzheimer’s, Prion, and Parkinson’s Diseases and Amyotrophic Lateral Sclerosis). Chem. Rev..

[39] Kozlowski H., Luczkowski M., Remelli M., Valensin D. (2012). Copper, zinc and iron in neurodegenerative diseases (Alzheimer’s, Parkinso’s and prion diseases). Coord. Chem. Rev..

[40] Hecel A., de Ricco R., Valensin D. (2016). Influence of membrane environments and copper ions on the structural features of amyloidogenic proteins correlated to neurodegeneration. Coord. Chem. Rev..

[41] Atrian-Blasco E., Gonzalez P., Santoro A., Alies B., Faller P., Hureau C. (2018). Cu and Zn coordination to amyloid peptides: From fascinating chemistry to debated pathological relevance. Coord. Chem. Rev..

[42] Hureau C. (2012). Coordination of redox active metal ions to the amyloid precursor protein and to amyloid-β peptides involved in Alzheimer disease. Part 1: An overview. Coord. Chem. Rev..

[43] Drew S.C., Barnham K. (2011). The Heterogeneous Nature of Cu^2+^ Interactions with Alzheimer’s Amyloid-β Peptide. Acc. Chem. Res..

[44] Migliorini C., Porciatti E., Luczkowski M., Valensin D. (2012). Structural characterization of Cu^2+^, Ni^2+^ and Zn^2+^ binding sites of model peptides associated with neurodegenerative diseases. Coord. Chem. Rev..

[45] Rana M., Sharma A.K. (2019). Cu and Zn Interactions with Aβ Peptides: Consequence of Coordination on Aggregation and Formation of Neurotoxic Soluble Aβ Oligomers. Metallomics.

[46] Faller P., Hureau C. (2009). Bioinorganic Chemistry of Copper and Zinc Ions Coordinated to Amyloid-Beta Peptide. Dalton Trans..

[47] Esmieu C., Guettas D., Conte-Daban A., Sabater L., Faller P., Hureau C. (2019). Copper-Targeting Approaches in Alzheimer’s Disease: How to Improve the Fallouts Obtained From in Vitro Studies. Inorg. Chem..

[48] Stefaniak E., Bal W. (2019). CuII Binding Properties of N-Truncated Aβ Peptides: In Search of Biological Function. Inorg. Chem..

[49] Bacchella C., Gentili S., Bellotti D., Quartieri E., Draghi S., Baratto M.C., Remelli M., Valensin D., Monzani E., Nicolis S. (2020). Binding and Reactivity of Copper to R 1 and R 3 Fragments of Tau Protein. Inorg. Chem..

[50] de Ricco R., Potocki S., Kozlowski H., Valensin D. (2014). NMR investigations of metal interactions with unstructured soluble protein domains. Coord. Chem. Rev..

[51] de Gregorio G., Biasotto F., Hecel A., Luczkowski M., Kozlowski H., Valensin D. (2019). Structural Analysis of copper(I) Interaction with Amyloid β Peptide. J. Inorg. Biochem..

[52] Giampietro R., Spinelli F., Contino M., Colabufo N.A. (2018). The Pivotal Role of Copper in Neurodegeneration: A New Strategy for the Therapy of Neurodegenerative Disorders. Mol. Pharm..

[53] Savelieff M.G., Nam G., Kang J., Lee H.J., Lee M., Lim M. (2019). Development of Multifunctional Molecules as Potential Therapeutic Candidates for Alzheimer’s Disease, Parkinson’s Disease, and Amyotrophic Lateral Sclerosis in the Last Decade. Chem. Rev..

[54] Nesi G., Sestito S., Digiacomo M., Rapposelli S. (2017). Oxidative Stress, Mitochondrial Abnormalities and Proteins Deposition: Multitarget Approaches in Alzheimer’s Disease. Curr. Top. Med. Chem..

[55] Tegoni M., Valensin D., Loso L., Remelli M. (2014). Copper Chelators: Chemical Properties and Bio-Medical Applications. Curr. Med. Chem..

[56] Valensin D., Gabbiani C., Messori L. (2012). Metal compounds as inhibitors of β-amyloid aggregation. Perspectives for an innovative metallotherapeutics on Alzheimer’s disease. Coord. Chem. Rev..

[57] Piemontese L., Vitucci G., Catto M., Laghezza A., Rullo F.M.P.M., Loiodice F., Capriati V., Solfrizzo M. (2018). Natural Scaffolds with Multi-Target Activity for the Potential Treatment of Alzheimer’s Disease. Molecules.

[58] Alkam T., Nitta A., Mizoguchi H., Itoh A., Nabeshima T. (2007). A Natural Scavenger of Peroxynitrites, Rosmarinic Acid, Protects Against Impairment of Memory Induced by Abeta(25–35). Behav. Brain Res..

[59] Taguchi R., Hatayama K., Takahashi T., Hayashi T., Sato Y., Sato D., Ohta K., Nakano H., Seki C., Endo Y. (2017). Structure-activity Relations of Rosmarinic Acid Derivatives for the Amyloid β Aggregation Inhibition and Antioxidant Properties. Eur. J. Med. Chem..

[60] Hase T., Shishido S., Yamamoto S., Yamashita R., Nukima H., Taira S., Toyoda T., Abe K., Hamaguchi T., Ono K. (2019). Rosmarinic Acid Suppresses Alzheimer’s Disease Development by Reducing Amyloid β Aggregation by Increasing Monoamine Secretion. Sci. Rep..

[61] Ono K., Hasegawa K., Naiki H., Yamada M. (2004). Curcumin Has Potent Anti-Amyloidogenic Effects for Alzheimer’s Beta-Amyloid Fibrils in Vitro. J. Neurosci. Res..

[62] Hamaguchi T., Ono K., Murase A., Yamada M. (2009). Phenolic Compounds Prevent Alzheimer’s Pathology Through Different Effects on the Amyloid-Beta Aggregation Pathway. Am. J. Pathol..

[63] Ono K., Li L., Takamura Y., Yoshiike Y., Zhu L., Han F., Mao X., Ikeda T., Takasaki J., Nishijo H. (2012). Phenolic Compounds Prevent Amyloid β-Protein Oligomerization and Synaptic Dysfunction by Site-Specific Binding. J. Biol. Chem..

[64] Iuvone T., de Filippis D., Esposito G., D′Amico A., Izzo A.A. (2006). The Spice Sage and Its Active Ingredient Rosmarinic Acid Protect PC12 Cells from Amyloid-Beta Peptide-Induced Neurotoxicity. J. Pharmacol. Exp. Ther..

[65] Rong H., Liang Y., Niu Y. (2018). Rosmarinic Acid Attenuates β-Amyloid-Induced Oxidative Stress via Akt/GSK-3β/Fyn-mediated Nrf2 Activation in PC12 Cells. Free Radic. Biol. Med..

[66] Hwang T.L., Shaka A.J.J. (1995). Water suppression that works. Excitation sculpting using arbitrary waveforms and pulsed field gradients. J. Magn. Reson. Ser. A.

[67] Akoury E. (2017). Isolation and Structural Elucidation of Rosmarinic Acid by Nuclear Magnetic Resonance Spectroscopy. Am. Res. J. Chem..

[68] Bertini I., Luchinat C. (1996). NMR of Paramagnetic Substances. Coord. Chem. Rev..

[69] Solomon I. (1955). Relaxation Processes in a System of Two Spins. Phys. Rev..

[70] ISO (2009). ISO 10995-5:2009, Biological evaluation of medical devices—Part 5: Tests for cytotoxicity: In vitro methods.

[71] Lamponi S., Leone G., Consumi M., Nelli N., Magnani A. (2020). Porous multi-layered composite hydrogel as cell substrate for in vitro culture of chondrocytes. Int. J. Polym. Mater. Polym. Biomater..

[72] Peisach J., Blumberg W.E. (1974). Structural Implications Derived From the Analysis of Electron Paramagnetic Resonance Spectra of Natural and Artificial Copper Proteins. Arch. Biochem. Biophys..

[73] Bennett B., Kowalski J.M. (2015). EPR Methods for Biological Cu(II): L-Band CW and NARS. Methods Enzymol..

[74] Danafetal N.A. (2016). Photophysical properties of neutral and dissociated forms of rosmarinic acid. J. Lumin..

[75] Valensin D., Gajda K., Gralka E., Valensin G., Kamysz W.H. (2010). Kozlowski Copper Binding to Chicken and Human Prion Protein Amylodogenic Regions: Differences and Similarities Revealed by Ni^2+^ as a Diamagnetic Probe. J. Inorg. Biochem..

[76] Valensin D., Camponeschi F., Luczkowski M., Baratto M.C., Remelli M., Valensin G., Kozlowski H. (2011). The Role of His-50 of α-Synuclein in Binding Cu(II): pH Dependence, Speciation, Thermodynamics and Structure. Metallomics.

[77] Remelli M., Ceciliato C., Guerrini R., Kolkowska P., Krzywoszynska K., Salvadori S., Valensin D., Watly J., Kozlowski H. (2016). DOES Hemopressin Bind Metal Ions in Vivo?. Dalton Trans..

[78] Remelli M., Valensin D., Toso L., Gralka E., Guerrini R., Marzola E., Kozłowski H. (2012). Thermodynamic and Spectroscopic Investigation on the Role of Met Residues in Cu(II) Binding to the Non-Octarepeat Site of the Human Prion Protein. Metallomics.

[79] Airoldi C., Sironi E., Dias C., Marcelo F., Martins A., Rauter A.P., Nicotra F., Jimenez-Barbero J. (2013). Natural Compounds Against Alzheimer’s Disease: Molecular Recognition of Aβ1-42 Peptide by Salvia Sclareoides Extract and Its Major Component, Rosmarinic Acid, as Investigated by NMR. Chem. Asian J..

[80] Shenberger Y., Marciano O., Gottlieb H.E., Ruthsteinet S. (2018). Insights into the N-terminal Cu(II) and Cu(I) binding sites of the human copper transporter CTR1. J. Coord. Chem..

[81] Rasia R., Bertoncini C.W., Marsh D., Hoyer W., Cherny D., Zweckstetter M., Griesinger C., Jovin T.M., Fernández C.O. (2005). Structural Characterization of copper(II) Binding to Alpha-Synuclein: Insights Into the Bioinorganic Chemistry of Parkinson’s Disease. Proc. Natl. Acad. Sci. USA.

[82] Kowalik-Jankowska T., Ruta M., Wiśniewska K., Lankiewicz L. (2003). Coordination Abilities of the 1–16 and 1–28 Fragments of Beta-Amyloid Peptide Towards copper(II) Ions: A Combined Potentiometric and Spectroscopic Study. J. Inorg. Biochem..

[83] Karr J.W., Kaupp L.J., Szalai V.A. (2004). Amyloid-beta Binds Cu^2+^ in a Mononuclear Metal Ion Binding Site. J. Am. Chem. Soc..

[84] Guilloreau L., Damian L., Coppel Y., Mazarguil H., Winterhalter M., Faller P. (2006). Structural and Thermodynamical Properties of CuII amyloid-beta16/28 Complexes Associated with Alzheimer’s Disease. J. Biol. Inorg. Chem..

