# Supplementary material for: Novel Perspective on Alzheimer’s Disease Treatment: Rosmarinic Acid Molecular Interplay with Copper(II) and Amyloid β
Source: Life (Basel). 2020 Jul 20;10(7):118. doi: 10.3390/life10070118 (PMC7400086; doi:10.3390/life10070118)
Supplement: Supplementary file 1 [file life-10-00118-s001.pdf]

life

Supplementary Material

Novel Perspective on Alzheimer's Disease Treatment: Rosmarinic Acid Molecular Interplay with Copper(II) and Amyloid β

Arian Kola [1], Aleksandra Hecel [2], Stefania Lamponi [1] and Daniela Valensin [1,*]

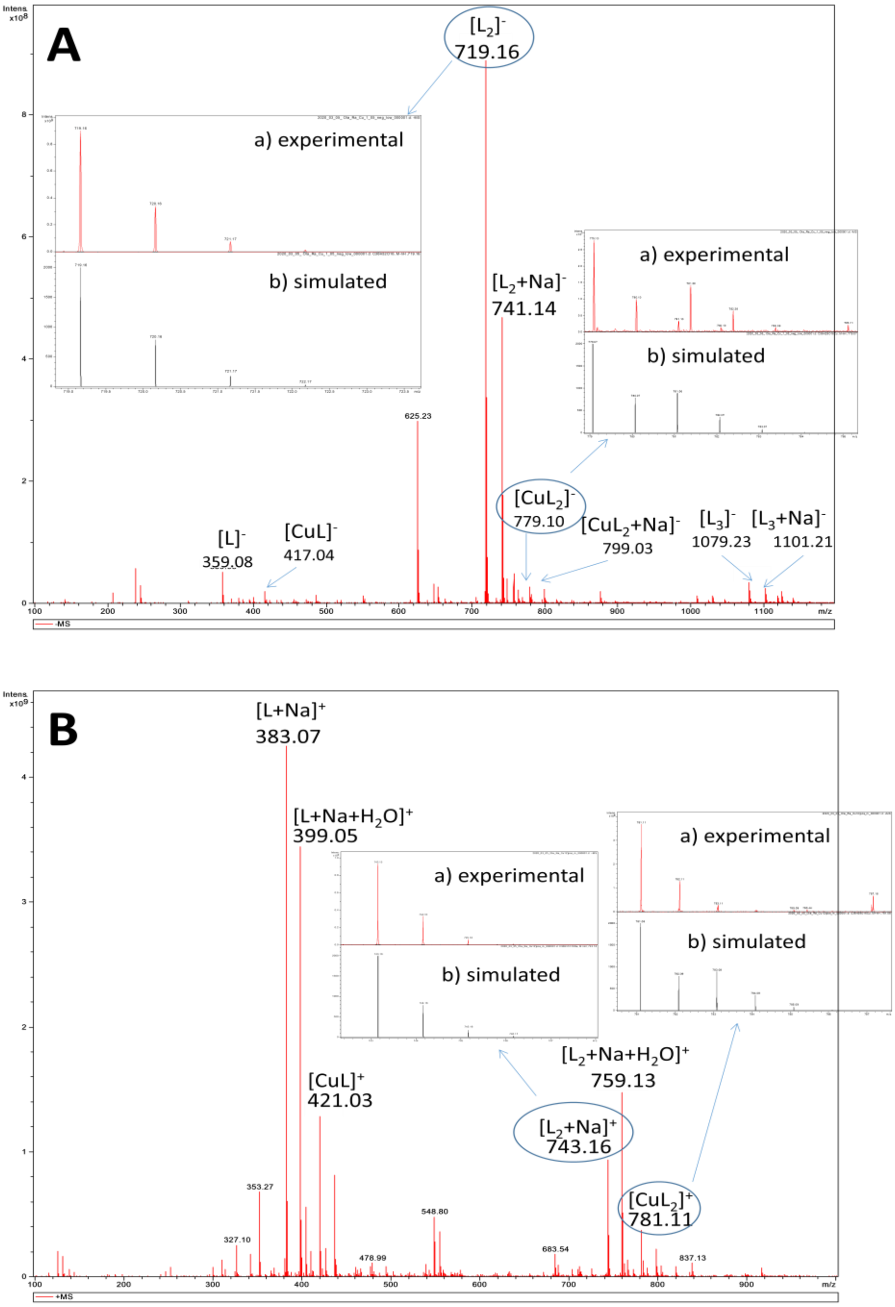

Figure S1. ESI-MS spectra of Cu(II)–RA system recorded in (**A**) negative and (**B**) positive ion mode. Molar ratio M:L 1:2; [L] = 5.0 × 10^{-4} M. The complex was prepared in a mixture of MeOH:H_2O (1:1) at

pH 7. For chosen complexes, a comparison of the experimental a) and simulated b) signals was performed.

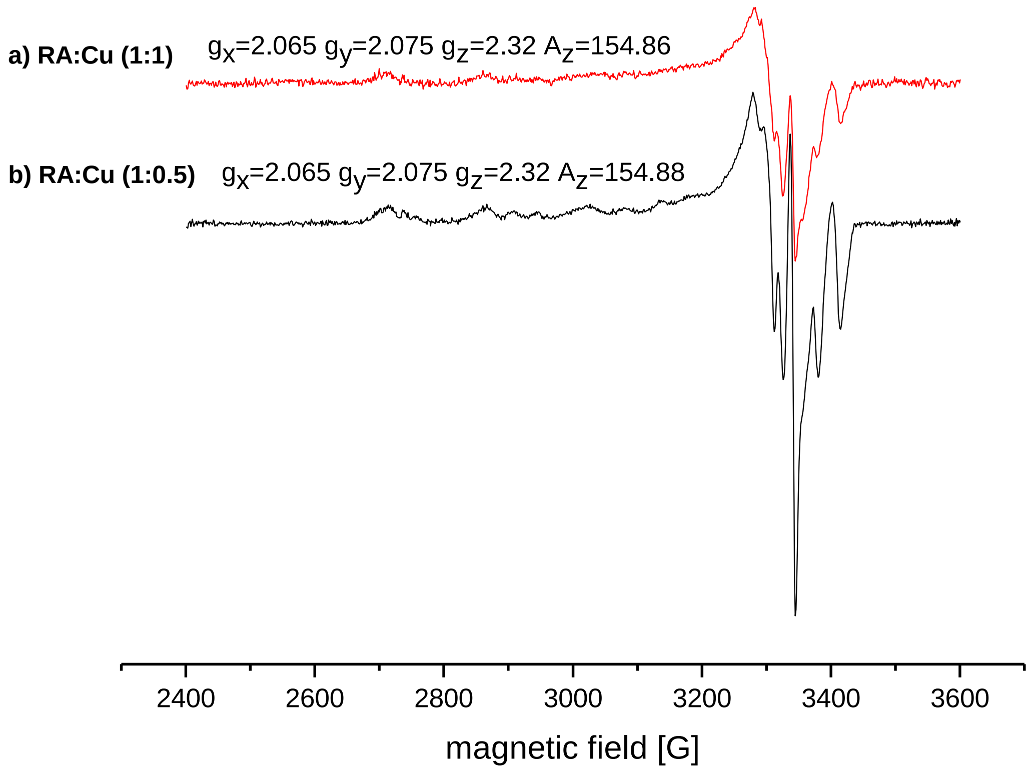

Figure S2. EPR spectra of RA–Cu(II) system in phosphate buffer (20% of ethylene glycol) at pH 7.4. T = 77K (X-band – 9.5 GHz). [Cu(II)] = 1.0×10^{-3} M, molar ratio M:L a) 1:1 and b) 0.5:1.

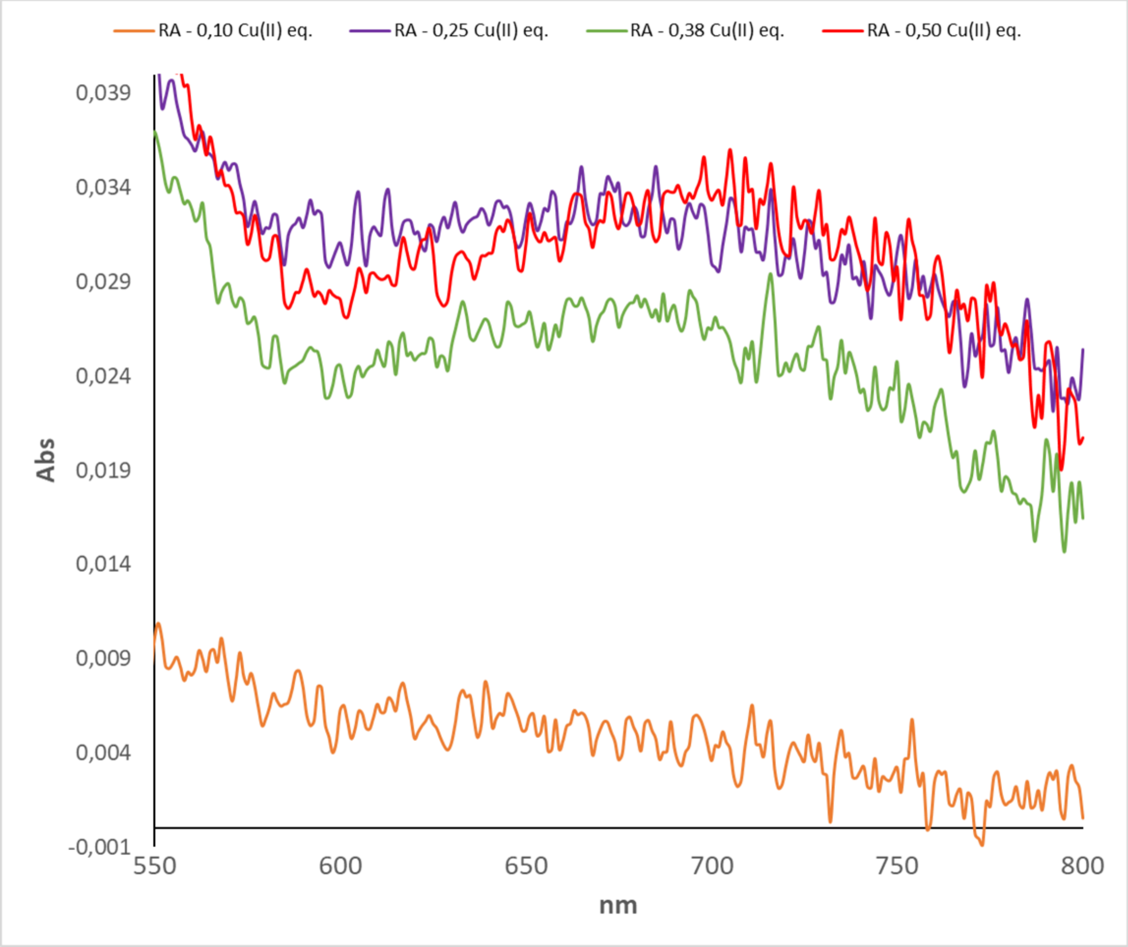

Figure S3. UV–VIS spectra (d–d transitions) of RA at pH 7.4 in presence of increasing Cu(II) concentrations. [RA] = 2.0×10^{-3} M; [phosphate buffer] = 1.8×10^{-2} M.

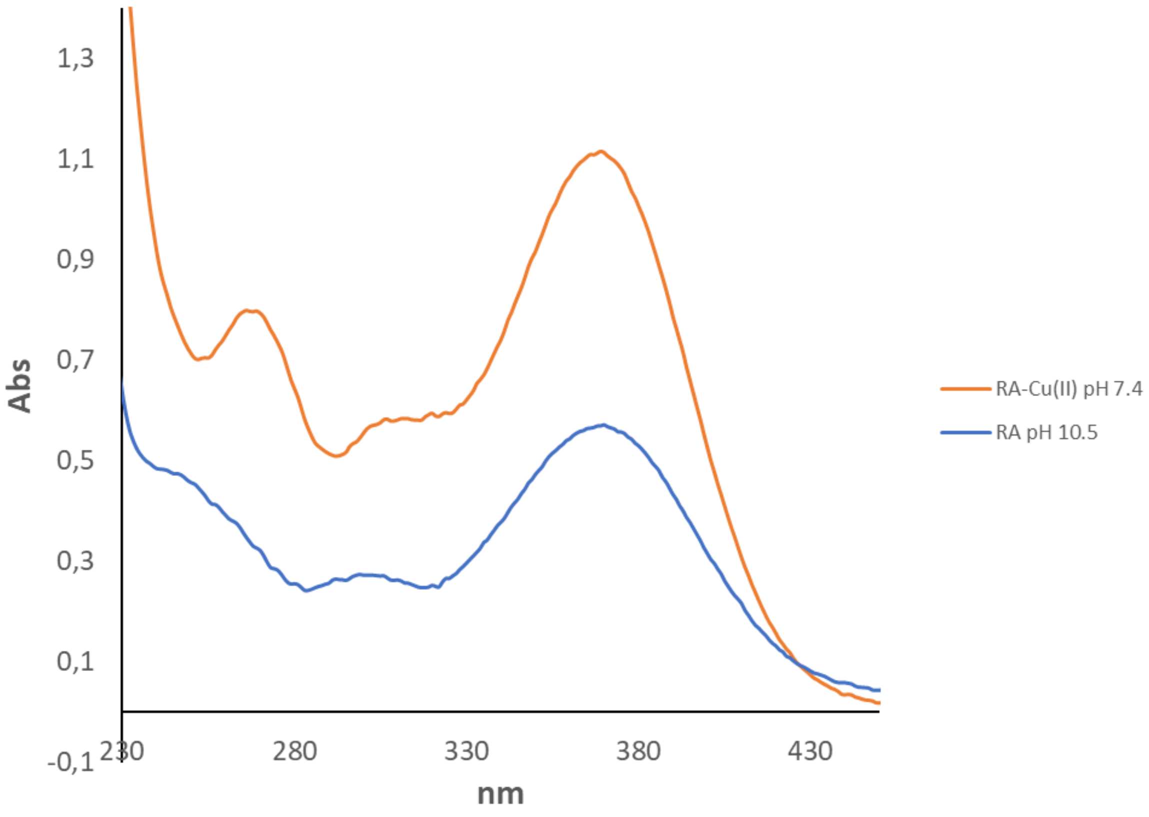

Figure S4. Comparison between UV–VIS spectra of RA–Cu(II) complex at pH 7.4 and of RA at basic pH. [RA] = 5.0×10^{-4} M; [Cu(II)] = 2.5×10^{-4} M; [phosphate buffer] = 1.8×10^{-2} M.

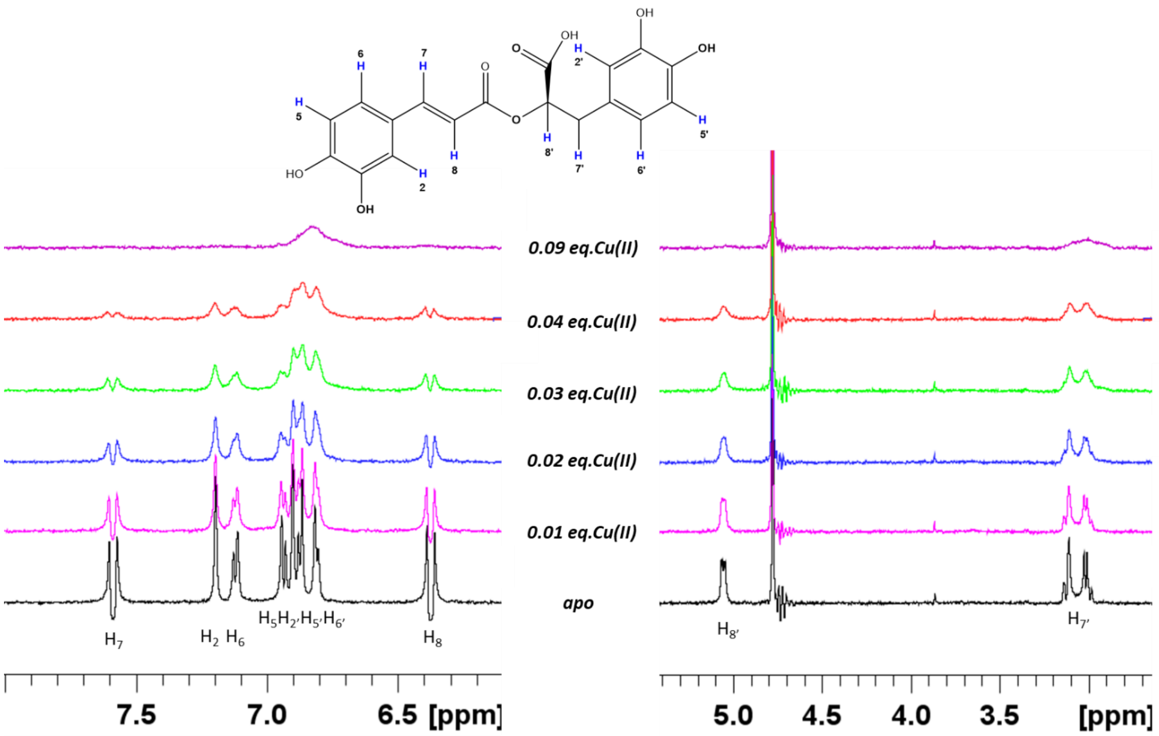

Figure S5. Superimposition of ^{1}H NMR spectra in absence (apo, black line) and in presence of increasing concentration of Cu(II) ions (colored lines). [RA] = 5.0×10^{-4} M; [phosphate buffer] = 1.8×10^{-2} M.

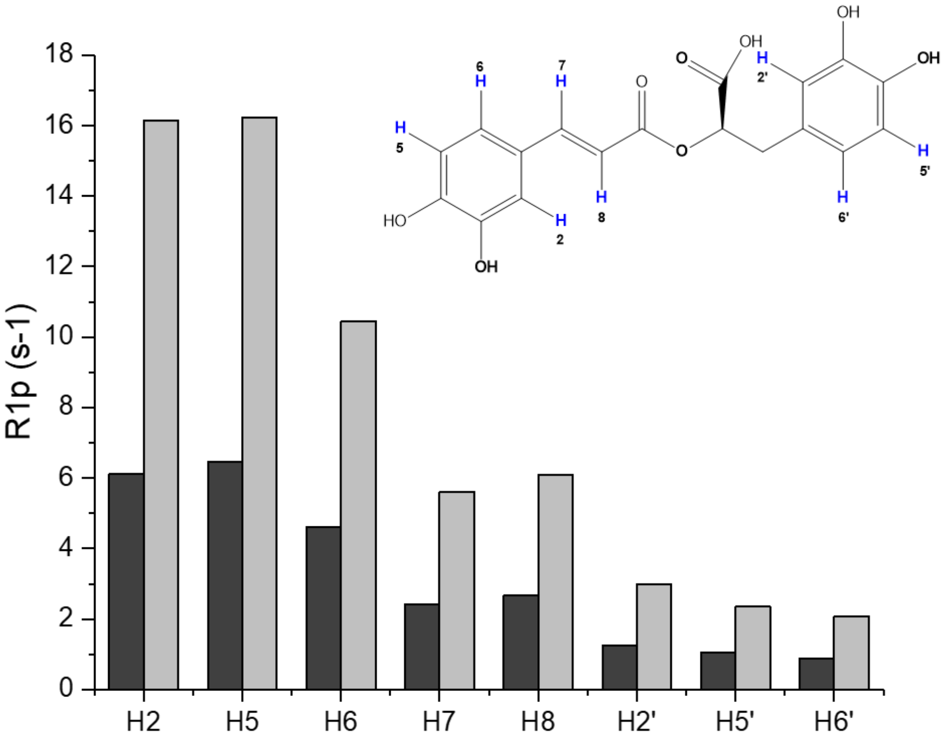

Figure S6. R_{1p} values of RA protons measured in solutions with 0.01 Cu(II) eqs. (dark grey) and 0.02 Cu(II) eqs. (light grey). [RA] = 5.0×10^{-4} M; [phosphate buffer] = 1.8×10^{-2} M.

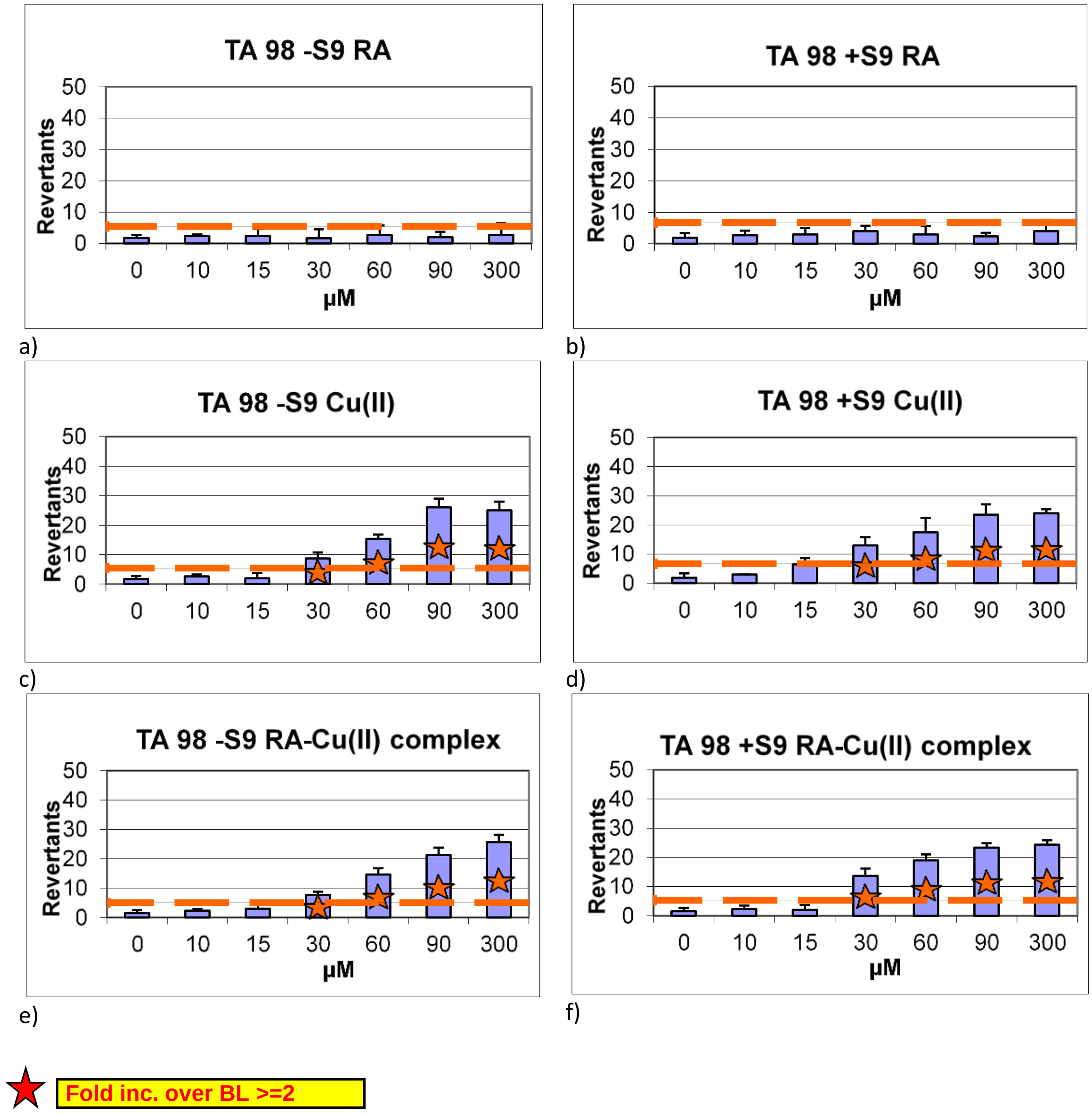

Figure S7. Ames test performed on *S. typhimurium* strain TA98, with and without S9 fraction, for RA (**a**, **b**), Cu(II) (**c**, **d**) and RA–Cu(II) complex (**e**, **f**). The Cu(II) concentration in the RA–Cu(II) complex was 10 µM for each RA concentration(10, 15, 30, 60, 90, and 300 µM).

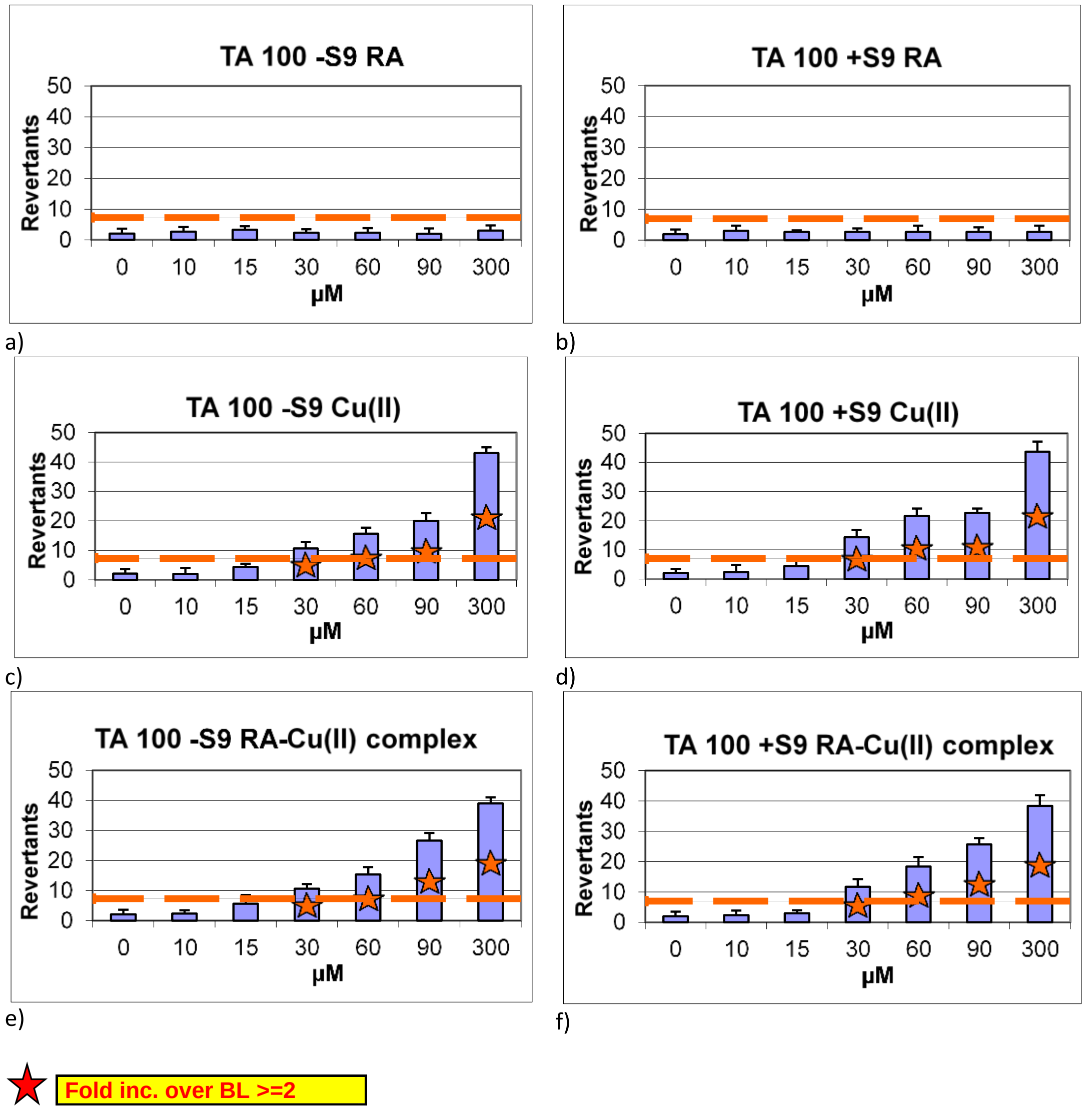

Figure S8. Ames test performed on *S. typhimurium* strain TA100, with and without S9 fraction, for RA (**a**, **b**), Cu(II) (**c**, **d**) and RA–Cu(II) complex (**e**, **f**). The Cu(II) concentration in the RA–Cu(II) complex was 10 µM for each RA concentration (10, 15, 30, 60, 90, and 300 µM).

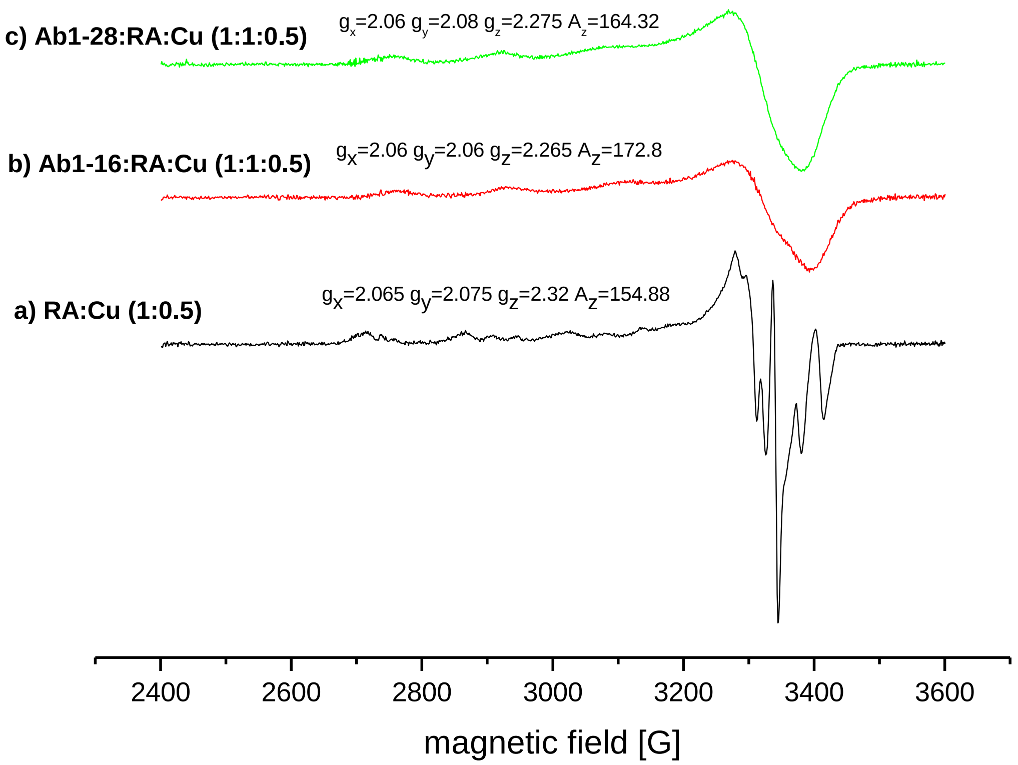

Figure S9. EPR spectra of (**a**) RA–Cu(II), (**b**) Aβ16–RA–Cu(II), (**c**) Aβ28–RA–Cu(II) systems in phosphate buffer (20% of ethylene glycol) at pH 7.4. T = 77K (X-band – 9.5 GHz). [Cu(II)] = 1.0 × 10^{-3} M; [RA] = [Aβ] = 2.0 × 10^{-3} M; [phosphate buffer] = 1.8 × 10^{-2} M.

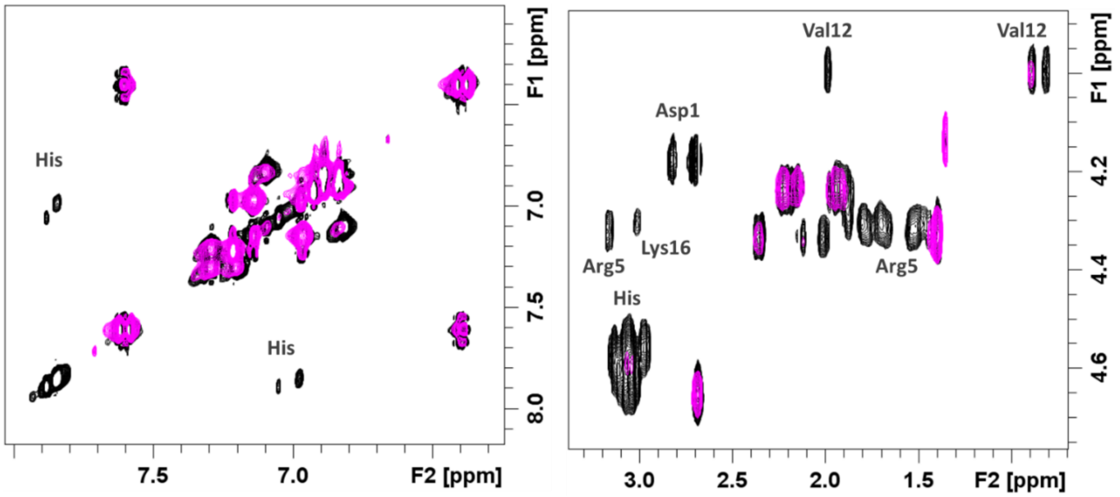

Figure S10. Superimposition of 2D NMR ^{1}H-^{1}H TOCSY of Aβ16–RA (black contours) and Aβ16–RA–Cu(II) (magenta contours) systems. [RA] = [Aβ] = 1.0 × 10^{-3} M; [Cu(II)] = 4.0 × 10^{-5} M; [phosphate buffer] = 1.8 × 10^{-2} M.

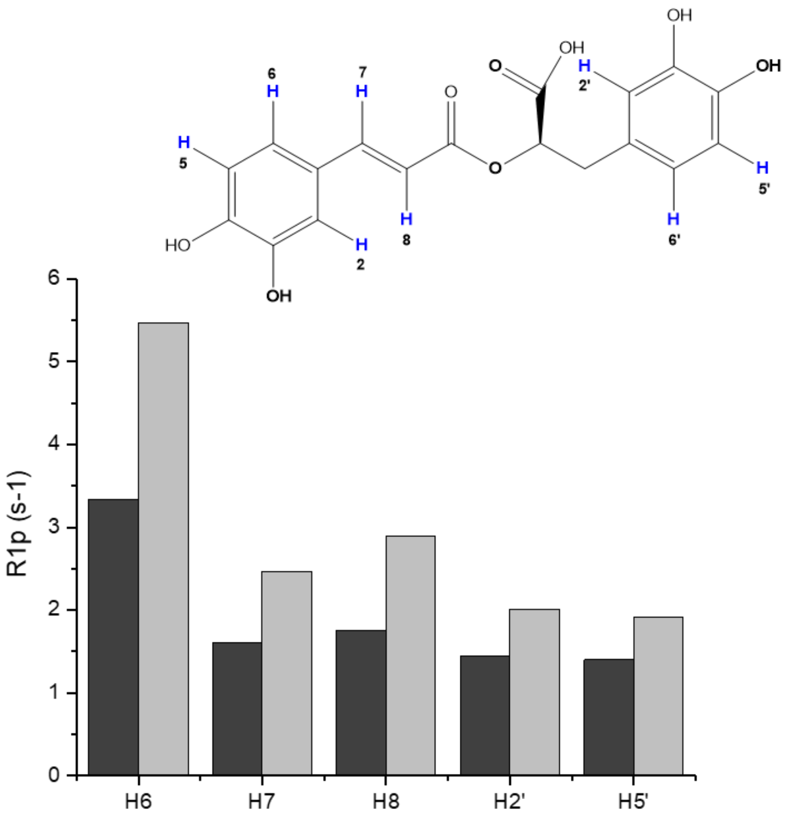

Figure S11. R_{1p} values of RA protons measured in Aβ16–RA solutions with 0.02 Cu(II) eqs. (dark grey) and 0.04 Cu(II) eqs. (light grey). [RA] = [Aβ] = 5.0×10^{-4} M; [phosphate buffer] = 1.8×10^{-2} M.

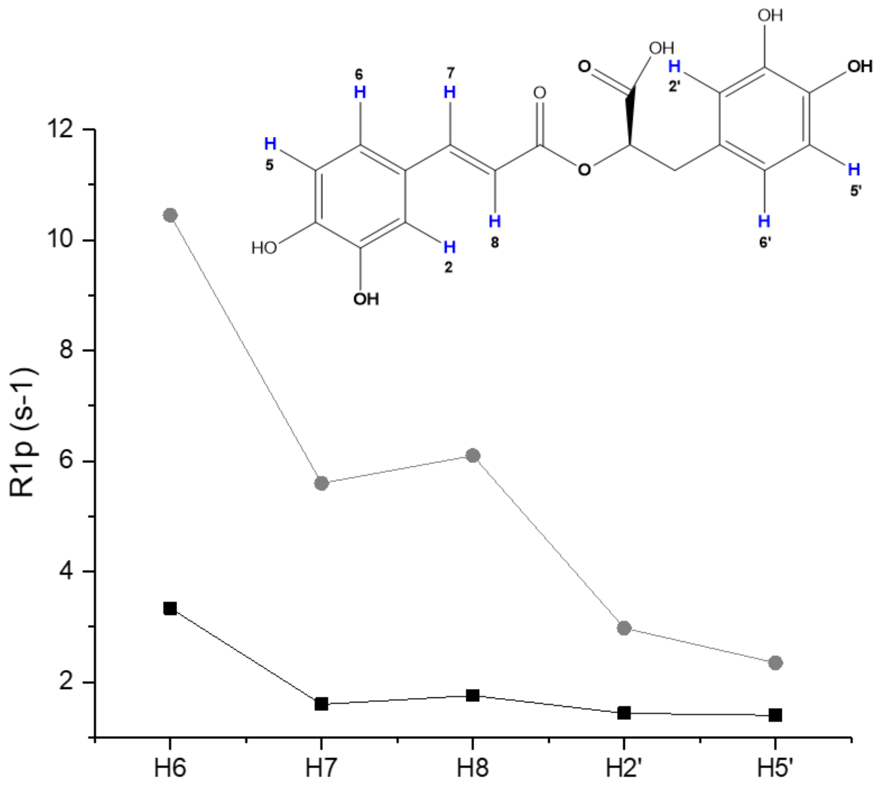

Figure S12. Comparisons between R_{1p} values of RA protons measured for Aβ16–RA–Cu(II) (black squares) and RA–Cu(II) (grey circles) solutions. [RA] = [Aβ] = 5.0 × 10^{-4} M; [Cu(II)] = 1.0 × 10^{-5} M; [phosphate buffer] = 1.8 × 10^{-2} M.
